# Strong increase in the autofluorescence of cells signals struggle for survival

**DOI:** 10.1038/s41598-018-30623-2

**Published:** 2018-08-14

**Authors:** Jérémy Surre, Claude Saint-Ruf, Valérie Collin, Sylvain Orenga, Mahendrasingh Ramjeet, Ivan Matic

**Affiliations:** 1 Institut National de la Santé et de la Recherche Médicale (INSERM) U1001, Université Paris Descartes, Sorbonne Paris Cité, Faculté de Médecine Paris Descartes, Paris, France; 2 bioMérieux SA, Microbiology Unit, R&D Microbiology, La Balme les Grottes, France; 3 INSERM U1016, Centre National de la Recherche Scientifique (CNRS) UMR8104, Institut Cochin, Paris, France; 4 CNRS, 75016 Paris, France

## Abstract

Prokaryotic and eukaryotic cells exhibit an intrinsic natural fluorescence due to the presence of fluorescent cellular structural components and metabolites. Therefore, cellular autofluorescence (AF) is expected to vary with the metabolic states of cells. We examined how exposure to the different stressors changes the AF of *Escherichia coli* cells. We observed that bactericidal treatments increased green cellular AF, and that *de novo* protein synthesis was required for the observed AF increase. Excitation and emission spectra and increased expression of the genes from the flavin biosynthesis pathway, strongly suggested that flavins are major contributors to the increased AF. An increased expression of genes encoding diverse flavoproteins which are involved in energy production and ROS detoxification, indicates a cellular strategy to cope with severe stresses. An observed increase in AF under stress is an evolutionary conserved phenomenon as it occurs not only in cells from different bacterial species, but also in yeast and human cells.

## Introduction

All prokaryotic and eukaryotic cells exhibit an intrinsic natural fluorescence (autofluorescence; AF) due to the presence of different fluorescent cellular structural components and metabolites, such as flavins, nicotinamide-adenine dinucleotide (NAD), aromatic amino acids, lipofuscins, advanced glycation end products, and collagen^1,2^. Cellular AF spectra encompass most of the spectral range because different endogenous fluorophores emit at different wavelengths of the electromagnetic spectrum. For example, flavins, NAD, and lipofuscin emit green, blue, and orange light respectively when excited at appropriate wavelengths. For this reason, AF frequently overlaps with the spectrum of exogenous fluorophores used for research purposes, and therefore interferes with the fluorescent microscopy and cytometric analyses. For example, AF precludes the detection of weak signals from the fluorescent reporters for low-abundance proteins. Correction of this “contaminating” AF is problematic because it is frequently unevenly distributed within and between cells. In addition, because cellular extracts are frequently components of the growth media, growth media are also commonly autofluorescent. For these reasons, considerable efforts have been made to develop methods to deal with these nuisances, which are referred to as background fluorescence, noise, or spectral crosstalk^1,2^.

However, cellular AF itself presents several advantages and can therefore be used for various analytical purposes. Firstly, cellular AF can be monitored without the need for labor-intensive sample preparation involving external fluorophores. Therefore, potential chemical toxicity for the sample and the user, and nonspecific binding and interference with biomolecular functions are avoided. Secondly, AF can be examined *in situ* without disrupting complex structures like bacterial biofilms and multicellular eukaryotic tissues. Thirdly, because cellular AF varies with the cellular morphology as well as with the metabolic and pathological states of cells, it can be used for diagnostic purposes^2,3^. For example, the change in tissue AF is used for non-invasive, *in vivo*, detection of tumors^4^. The infection of human HeLa cells with enterohemorrhagic *Escherichia coli* was also monitored by measuring the change in the AF of HeLa cells^5^. AF was also used as a reliable biomarker of the senescence of *Caenorhabditis elegans* nematodes^6^. AF can also be used for rapid detection and identification of bacterial contaminants in food because different bacterial strains and species have distinct intrinsic fluorescence^7–13^.

Among different endogenous fluorophores, flavins and NAD are extensively studied because they are responsible for most of the cytoplasmic AF and because of their prominent role in cell metabolism. Flavins, which comprise a category of molecules involving riboflavin and its derivatives flavin adenine dinucleotide (FAD) and flavin mononucleotide (FMN), and NAD are involved in various redox reactions. For example, FAD and NAD, which emit fluorescence when they are oxidized and reduced, respectively^2^ play a key role in the conversion of energy from acetyl CoA to ATP. Therefore, AF derived from these molecules is expected to vary as a function of the ATP production in different cellular growth phases, and because of the variable nutrient availability and presence of stressors. FAD and FMN are also associated with proteins, some of which are included in the protection against reactive oxygen species (ROS)^14^.

In this study, we examined how AF of *E. coli* cells changes as a function of exposure to different stressors. As stressors, we used antibiotics that, besides their medical importance, are powerful tools for unraveling complexity of bacterial physiology^15^, and sodium hypochlorite (bleach) which is a widely used bactericidal agent. We used two classes of antibiotics: ß-lactams that are cell wall synthesis inhibitors, and the protein synthesis inhibitors tetracycline and gentamicin. We found that treatment of *E. coli* with the ß-lactam antibiotic ampicillin or with sodium hypochlorite, significantly increased cellular AF, while no significant AF increase was observed in protein synthesis inhibitor-treated cells. Our data suggest that flavins are major contributors to bactericidal treatment-induced AF and that AF increase reveals cellular adaptive response to cope with the life-threating stressors. Finally, we demonstrated that the increase in green cellular AF subjected to life-threating treatments is an evolutionary conserved phenomenon as it occurs not only in cells from different bacterial species but also in yeast and human cells.

## Results

### Ampicillin-induced increase of autofluorescence

**Figure 1:**
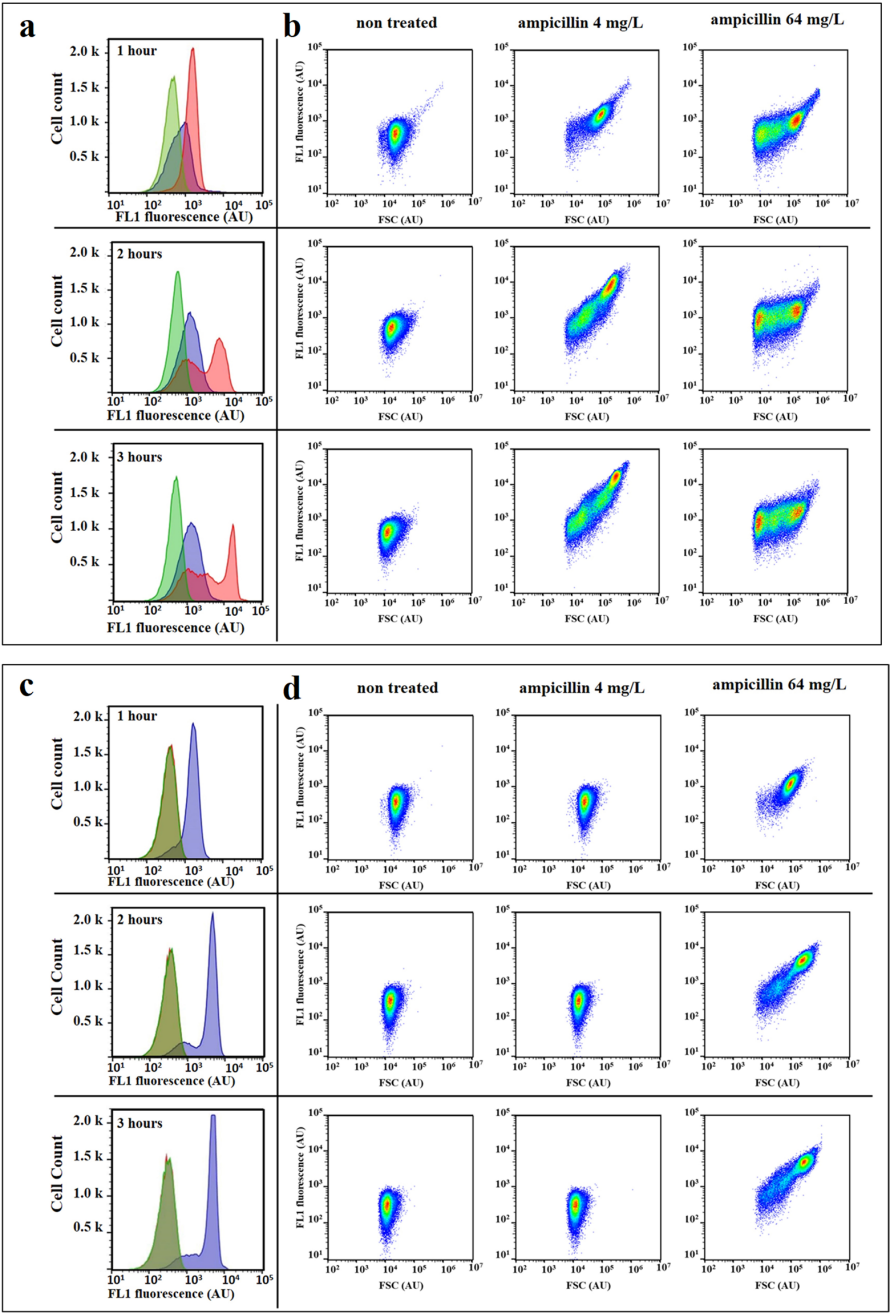
Effect of ampicillin treatment on the forward light scattering and autofluorescence intensity of *E. coli* cells. *E. coli* 7705035 [MIC = 4 mg/L; (**a**) and (**b**)] and 8812112 [MIC = 64 mg/L; (**c**) and (**d**)] cells were treated with a range of ampicillin concentrations for 3 hours of the exponential growth. Autofluorescence and forward light scattering signal (FSC) of treated cells and non-treated control were measured after 1, 2 and 3 hours of incubation using the Gallios flow cytometer. (**a**) and (**c**) The change in the distribution profiles of the cellular autofluorescence (λ_ex_ 488 nm/λ_em_ 525/20 nm) over time, for the ampicillin concentrations: 0 (green), 4 (red) and 64 mg/L (blue). (**b**) and (**d**) 2-dimensional representation of the FSC and FL1 (λ_ex_ 488 nm/λ_em_ 525/20 nm) of each event (n = 50,000) at 1, 2 and 3 hours of treatment with 0, 4, and 64 mg/L of ampicillin. This figure presents results of one representative experiment which was independently repeated three times. The overlap of green and red produces the khaki area.

We first tested whether treatment with the ß-lactam antibiotic ampicillin increases AF of *E. coli* cells. We used two *E. coli* strains which are susceptible (7705035) and resistant (8812112) to ampicillin according to the Clinical & Laboratory Standards Institute guidelines (www.clsi.org) (Table S1). Minimum inhibitory concentrations (MIC) of these strains are 4 mg/L and 64 mg/L respectively. Exponentially growing cultures of both strains were treated for 3 hours with a range of ampicillin concentrations. Cellular AF, as well as light scattering profiles, were analyzed by flow cytometry (Figs 1, S1 and S2). When compared to untreated conditions, all tested ampicillin concentrations induced an increase in green AF (excitation at 488 nm with bandpass filters of 530/30). Maximal AF was observed at ½ and 1× MIC. Of note, susceptible cells treated at 1× MIC revealed important heterogeneity in the autofluorescence distribution profile, which reflects cell-to-cell metabolic state heterogeneity. A similar trend was observed with the resistant strain. At concentrations above the MIC, cellular AF was higher than that of the untreated control, but lower than the AF measured at the MIC. Such an increase in the cellular AF, as a response to cytotoxic antibiotic treatment, was observed for other ampicillin-treated *E. coli* strains, but also for meropenem-treated *Klebsiella pneumoniae* and *Serratia marcescens* (Fig. S2).

The forward scattering (FSC) profiles of ampicillin-treated cells became dispersed, due to the appearance of cells of different shapes and sizes relative to the untreated control, and they changed over time (Figs 1 and S1). After one hour of incubation, cells treated with 1× and 16× MIC started increasing in size. After two hours of incubation, cells treated with 1× MIC continued to increase in size and smaller cells increased in frequency within populations of cells treated with 16× MIC. After three hours of incubation, smaller cells increased in frequency within populations of cells treated with both ampicillin concentrations. This could be explained by the mode of action of ß-lactam antibiotics that interfere with the synthesis of the peptidoglycan layer, which provides structural strength to the cell and balances osmotic pressure^16^. Consequently, treatment with the ß-lactams results in a loss of cell-wall integrity, elongation, bulge formation and ultimately cell lysis^17,18^. This raised the question of whether increased AF results from the increase in cell size upon antibiotic treatment, as was previously suggested^19^.

To address this question, we calculated the correlation coefficient r between the cell AF (FL1) and: (i) the FSC that is a proxy for cell size; (ii) the side scattering (SSC) that reflects cell internal complexity (Table S2). r values between FL1 and FSC increased over time for populations treated with the ampicillin concentrations below the MIC, while they decreased for the ampicillin concentration above the MIC. The same tendency was observed for the correlation between FL1 and the SSC.

However, when we normalized AF values of each individual cell with its FSC values, we observed that AF values increased significantly in *E. coli* cells treated with ≥MIC of ampicillin. The same phenomenon was observed for *K. pneumoniae* and *S. marcescens* treated with meropenem (Fig. S2). This result, combined with the correlation data between light scattering and cell fluorescence values (Table S2), suggests that the increase in AF is not due to the increase in bacterial cell size. This was observed for four different *E. coli* strains and two strains from other species, illustrating that the observed phenomena do not depend on a particular genetic background.

**Figure 2:**
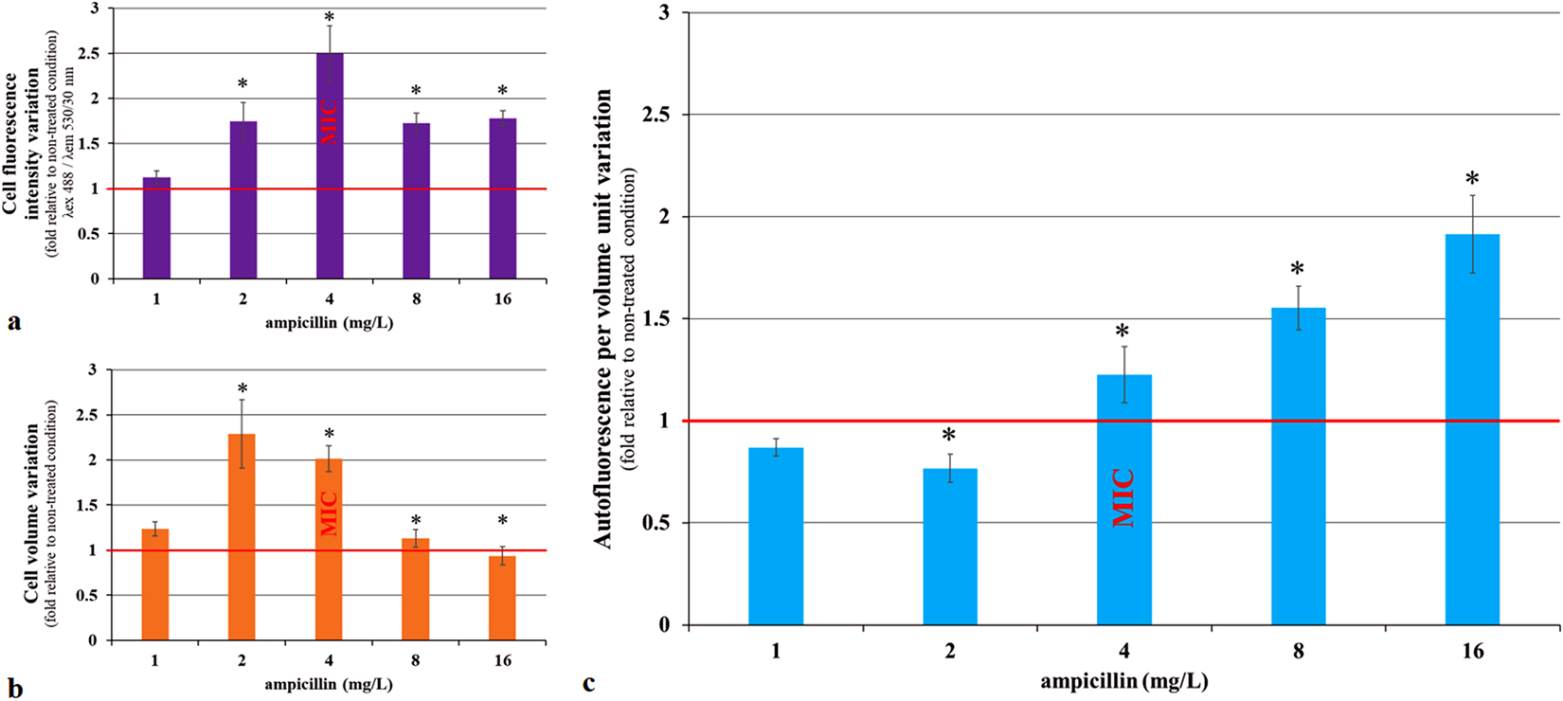
Change in the autofluorescence, volume and autofluorescence per volume unit of *E. coli* cells after 2 hours of ampicillin treatment. *E. coli* 7705035 cells were treated, with a range of ampicillin concentrations during exponential growth. After 2 hours of treatment, images of 20,000 cells per condition were collected using the ImageStream flow cytometer system. (**a**) The cell autofluorescence intensity (λ_ex_ 488 nm/λ_em_ 530/30 nm). (**b**) The cell volume. (**c**) The autofluorescence intensity per volume unit. For each cell, the fluorescence was normalized by the cell volume. Data are represented as a variation relative to a non-treated control. Each value represents the mean (+/−standard error) of the median values of four independent experiments. The asterisk represents significant differences in the ampicillin 1 versus ampicillin 0 (reference) condition according to 1-way ANOVA Dunn’s comparison test p value < 0.05.

**Figure 3:**
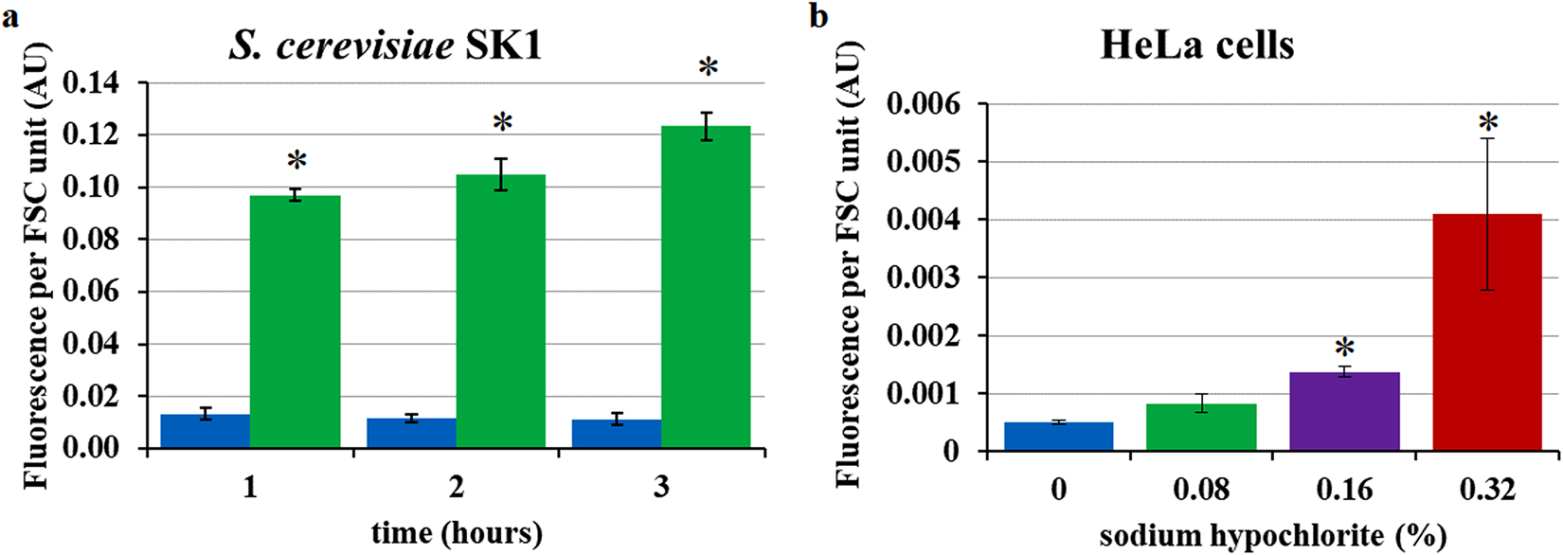
Sodium hypochlorite-induced autofluorescence increase in eukaryotic cells. Yeast and human cells were treated with sodium hypochlorite and their autofluorescence was assessed by flow cytometry (λ_ex_ 488/λ_em_ 525/20 nm). (**a)** *S. cerevisiae* SK1 strain. (**b**) HeLa human cervical cancer cells. Non-treated (blue). Sodium hypochlorite 0.08% (green), 0.16% (violet), 0.32% (red). *S. cerevisiae* was treated with 0.08% sodium hypochlorite for 1, 2 and 3 hours, while HeLa cells were treated with sodium hypochlorite for 1 hour. The asterisk represents significant differences relative to the non-treated controls according to the unpaired t-test; p value < 0.05. Each value represents the mean (+/−standard error) of the median values of three independent experiments.

Because the light scattering parameters only give approximate values of cell size and shape, we also analyzed ampicillin-treated *E. coli* (MIC 4 mg/L) cells using the ImageStream imaging flow cytometer system (Fig. 2). We collected images of 480,000 cells (20,000 cells for each of the six concentrations and four repetitions). We quantified the AF of each cell. We also calculated the volume of each cell using the following formula:

$${\rm{V}}=\frac{{\rm{\pi }}}{4}\cdot {{\rm{width}}}^{2}\cdot ({\rm{length}}-\frac{{\rm{width}}}{3}),$$

as previously described^20^. We observed that after 2 hours of incubation, both the AF intensity and the volume of cells treated with 0.25× and 0.5× MIC of ampicillin, increased in comparison to untreated controls. However, because the cell volume increased more than the AF, the AF normalized per cell volume decreased significantly (p value < 0.05 - One-way ANOVA Dunn’s comparison test). In cells treated with 1×, 2× and 4× MIC, the normalized AF increased significantly (p value < 0.05 - One-way ANOVA Dunn’s comparison test). The normalized AF increase was particularly strong at higher ampicillin concentrations because of the increased frequency of the small cells. Therefore, it can be concluded that an increase in the AF of ampicillin-treated bacterial cells is not an artifact due to increased cell size. Importantly, we also observed a significant increase in the AF of the yeast *Saccharomyces cerevisiae* and HeLa human cancer cells treated with a cytotoxic agent, sodium hypochlorite^21^ (Fig. 3). Taken together, our data indicate that AF increases in stressed prokaryotic and eukaryotic cells.

### Protein synthesis is a prerequisite for the increase in autofluorescence

**Figure 4:**
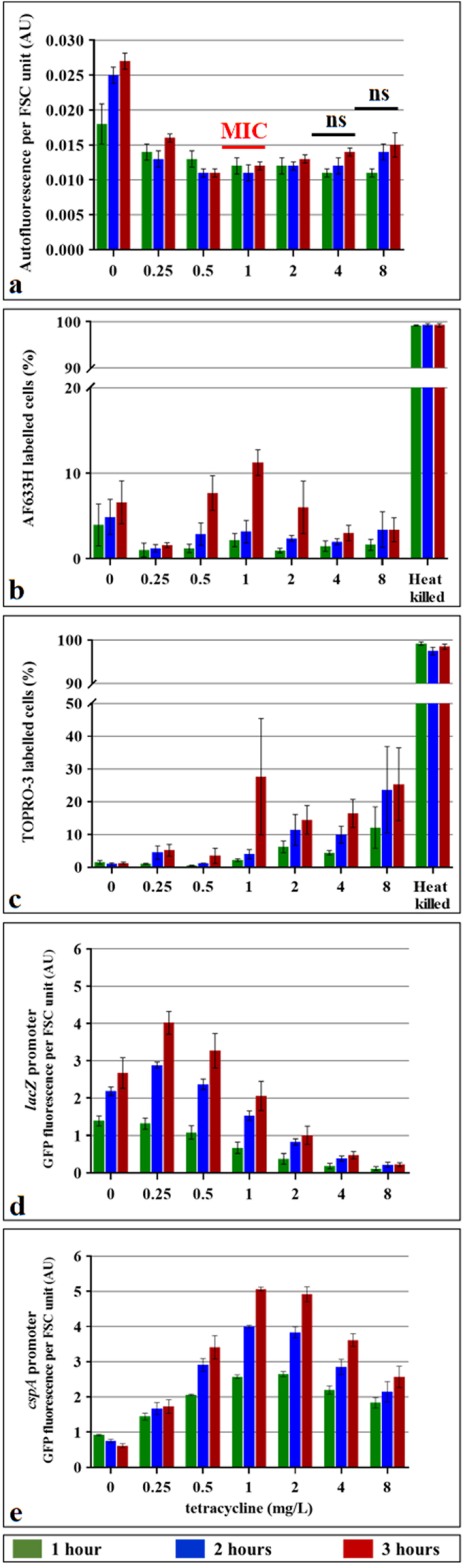
Effect of tetracycline treatment on the autofluorescence, protein synthesis and viability of *E. coli* cells. *E. coli* 7705035 cells were treated with a range of tetracycline concentrations for 3 hours of the exponential growth. The MIC of this strain is 1 mg/L of tetracycline. Autofluorescence (**a**) and cell viability were assessed using AF633H (**b**) and TOPRO-3 (**c**) dyes and flow cytometer. As a control for staining of dead cells, cells were killed by incubation at 65 °C for 30 min. The impact of tetracycline on protein synthesis was assessed using *lacZ* (**d**) and *cspA* (**e**) promoters which were fused to the gene coding for the fast folding green fluorescent protein (GFP). *cspA* is known to be induced by protein synthesis inhibiting antibiotics, while *lacZ* should be repressed. Exponentially growing *E. coli* cells carrying plasmids with these reporter fusions, as well as promoter-less control plasmid, were incubated with IPTG and a range of tetracycline concentrations for 3 hours. Background autofluorescence was removed by subtracting the promoter-less autofluorescence. GFP fluorescence and FSC of each cell was monitored with the flow cytometer and cell autofluorescence or GFP fluorescence was normalized by the FSC. Values represent the mean (+/−standard error) of the median values of three independent experiments.

Because treatment with bactericidal antibiotic ampicillin increases cellular AF, we tested the impact of a bacteriostatic antibiotic tetracycline on AF. Tetracycline inhibits bacterial protein synthesis by preventing the binding of aminoacyl-tRNA to ribosome-mRNA complex^22^. Therefore, tetracycline prevents cell growth but does not kill cells. Exponentially growing cultures of a sensitive *E. coli* strain were treated with a range of tetracycline concentrations for 3 hours. We did not observe increased AF in tetracycline-treated cells at any of the concentrations tested (Fig. 4).

To confirm that tetracycline did affect treated cells, we used two plasmid-borne reporters which consist of fusions of the promoters of the *cspA* and *lacZ* genes to a gene coding for green fluorescent protein (GFP)^23^. As expected, the expression of the *cspA* gene, which is known to be induced by treatment with antibiotics that inhibit protein synthesis^24^, increased upon tetracycline treatment (Fig. 4e). A peculiar structure of the *cspA* mRNA allows preferential translation, even in the absence of synthesis of other proteins due to antibiotic treatment or low temperature^25^. The *lacZ* promoter-driven GFP fluorescence decreased with increasing doses of tetracycline in spite of its induction with IPTG, which indicates that tetracycline blocks protein synthesis (Fig. 4). These experiments confirmed that tetracycline had affected treated cells but that this did not increase AF.

We also evaluated the viability of cells treated with tetracycline using the viability markers, Alexa Fluor™ 633 Hydrazide (AFH) which targets carbonyl groups, and TO-PRO™-3 which binds to DNA in cells with damaged membranes^26,27^. Both viability markers confirmed that tetracycline did not kill the cells (Fig. 4) but that they both stained heat-killed cells.

**Figure 5:**
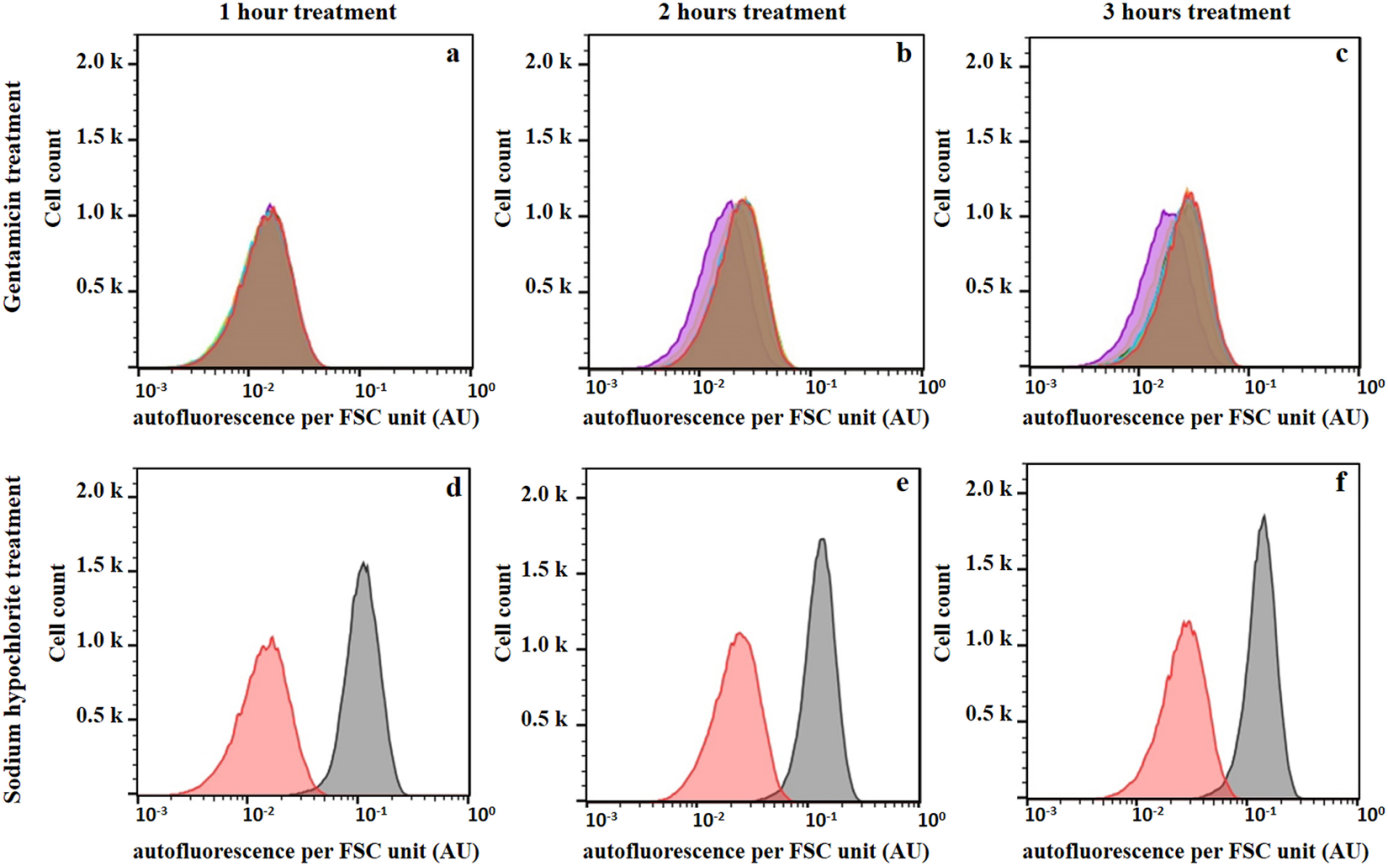
Effects of gentamicin and sodium hypochlorite treatment on the autofluorescence of *E. coli* cells. *E. coli* 7705035 cells were treated with a range of gentamicin concentrations corresponding to 0 (red), 0.125 (light blue), 0.25 (orange), 0.5 (MIC; light green), 1 (dark green), 2 (brown) and 4 mg/L (violet) or 0.08% sodium hypochlorite (grey) for 3 hours of the exponential growth. Autofluorescence and FSC of treated cells and untreated control were measured after 1, 2 and 3 hours of incubation using a flow cytometer. Cell autofluorescence was normalized by the FSC signal to obtain the autofluorescence per FSC unit. (**a)**, (**b)** and (**c**) 1, 2 and 3 hours of gentamicin treatment respectively. The overlap of red, light blue, orange, light green and dark green produces the brown area. (**d)**, (**e)** and (**f**) 1, 2 and 3 hours of sodium hypochlorite treatment respectively. This figure presents results of representative experiments, which were independently repeated 4 times.

This raised the question of whether AF did not increase in tetracycline-treated cells because they were alive or because tetracycline inhibited protein synthesis. To address this question, we treated cells with the bactericidal protein synthesis inhibitor gentamicin^28^ and with the bactericidal agent sodium hypochlorite (Fig. 5). We observed that gentamicin treatment did not increase cellular AF. However, sodium hypochlorite strongly increased cellular AF. Taken together, our data indicate that protein synthesis is a prerequisite for the increase in AF.

### Autofluorescence spectra correspond to flavin fluorescence

**Figure 6:**
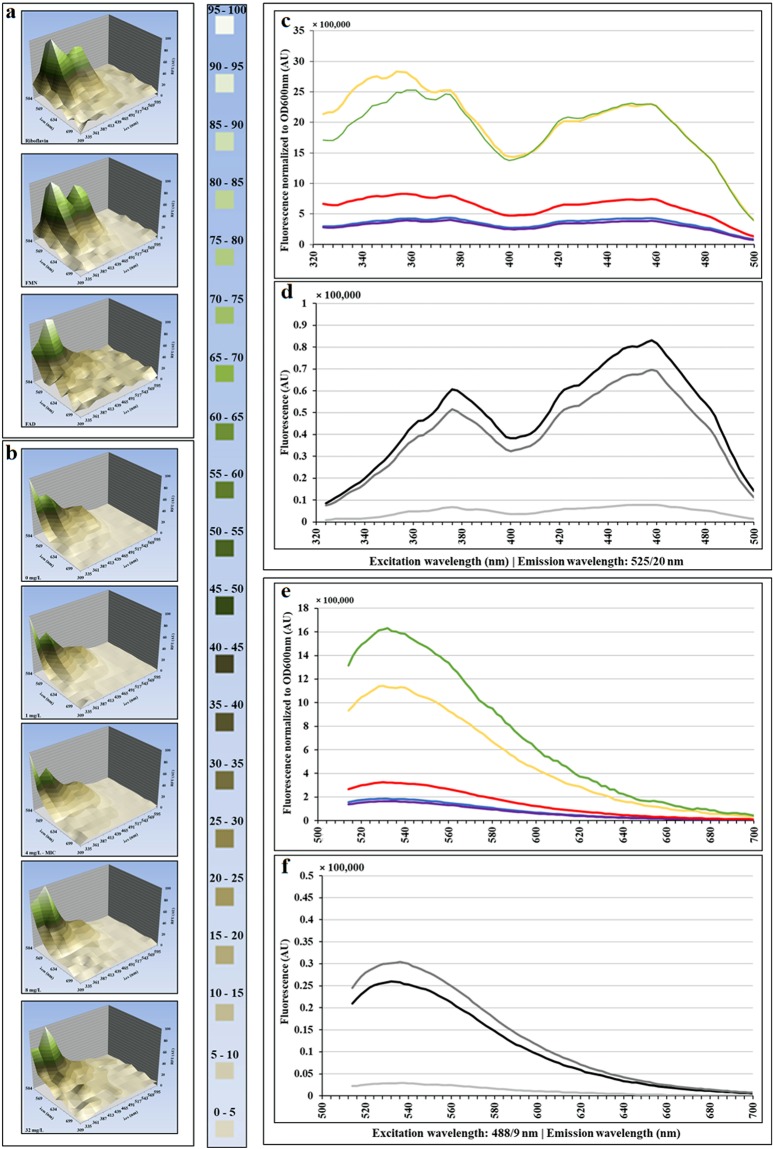
Comparison of excitation and emission spectra of ampicillin-treated *E. coli* cells and purified flavonoid compounds. *E. coli* 7705035 cells [MIC = 4 mg/L] growing exponentially in M9 medium supplemented with glucose, were treated with a range of ampicillin concentrations for 6 hours. After 6 hours of treatment, the excitation and emission spectra of treated cells were established and compared with the spectra of FAD, FMN and riboflavin. Three-dimensional representation of fluorescence spectra of flavins **(a)** and *E. coli* **(b)**. **(c)** and **(e)**: excitation and emission spectra of *E. coli* cells treated with ampicillin, normalized to the optical density at 600 nm. The colors represent ampicillin concentrations: 0 (blue line), 1 (violet line), 4 (red line), 8 (yellow line) and 32 mg/L (green line). **(d)** and **(f)**: excitation and emission spectra of 10 µM purified flavonoid compounds: FAD, FMN and riboflavin. The colors represent: light grey for FAD, black for FMN and dark grey for riboflavin. This figure presents results of representative experiments, which were independently repeated 4 times.

Different fluorescent cellular components and metabolites have different excitation and emission wavelengths^2,4^. We aimed to identify the endogenous fluorophores that are responsible for the observed increase in cell AF. Using several excitation and emission wavelength combinations, we investigated the fluorescence per FSC unit of ampicillin-treated *E. coli* by flow cytometry and fluorimetry. There were two peaks of excitation, one between 320 and 400 nm (maxima at 354 and 372 nm) and another between 408 and 488 nm (maximum at 458 nm, Fig. 6). The peak of emission was 525 nm (Fig. S3). These excitation and emission AF wavelengths correlated well with the flavin spectral properties^4^. The comparison of the excitation and emission AF spectra of cells treated with ampicillin in LB (Fig. S4) or in M9 medium (Fig. 6), with the spectra of purified flavins: riboflavin, FMN and FAD, further confirmed the correlation.

**Figure 7:**
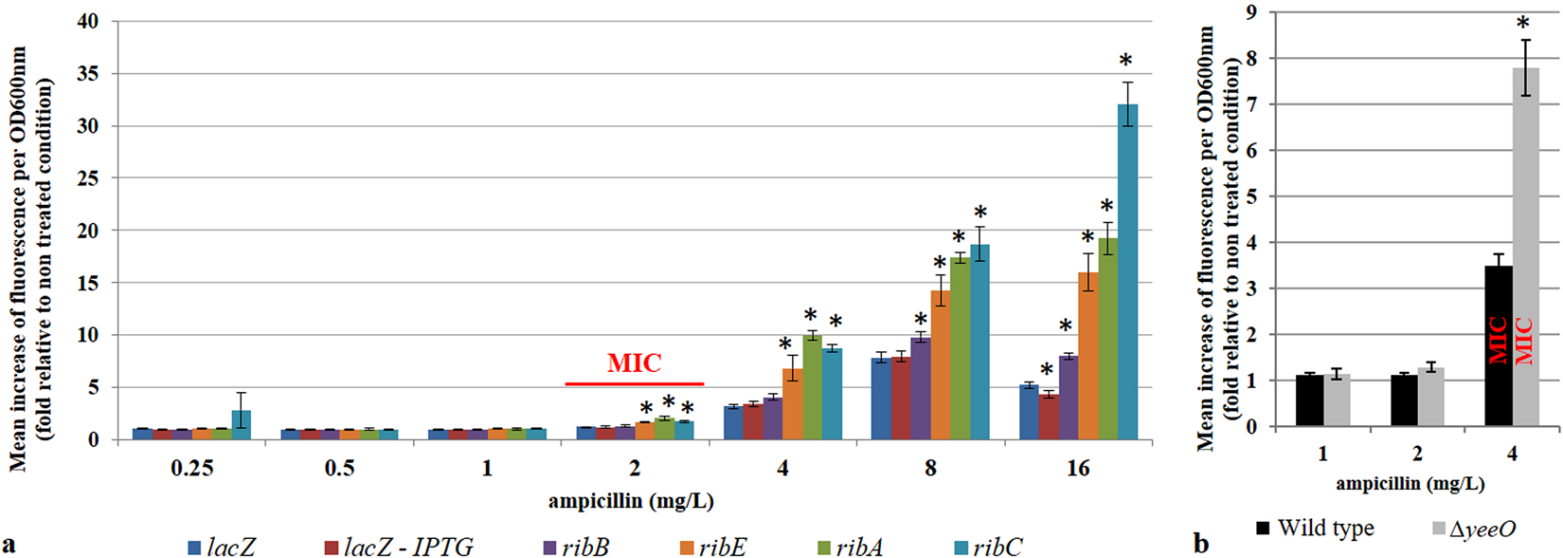
Expression of *rib* genes in ampicillin-treated *E. coli* cells. (**a**) The promoters of *lacZ* (dark blue), *ribA* (green), *ribB* (violet), *ribC* (light blue) and *ribE* (orange) genes were fused to the gene coding for the fast-folding green fluorescent protein (GFP). The promoter of the *lacZ* gene was induced with IPTG (red). *E. coli* MG1655 strains (MIC ampicillin = 2 mg/L) carrying plasmids with these transcription reporter fusions were grown with a range of ampicillin concentrations. After 3 hours of incubation, GFP fluorescence (λ_ex_ 488/9/λ_em_ 525/20 nm) and OD_600 nm_ of treated cells and non-treated control were measured using a fluorimeter. The fluorescence increase was calculated using the non-treated control of each strain as reference. (**b**) Wild type BW25113 strain and its ∆*yeeO* derivative were treated with a range of ampicillin concentrations during exponential growth. Autofluorescence (λ_ex_ 440/9/λ_em_ 525/20 nm) and OD_600nm_ of treated cells and non-treated controls were measured using a fluorimeter. To calculate the increase in the autofluorescence due to ampicillin treatment, autofluorescence of treated cells was first normalized by OD_600nm_ and then by the normalized autofluorescence of the non-treated controls of each strain. Each value represents the mean (+/−standard error) of the fluorescence increase from six independent experiments. The asterisk represents significant differences with the *lacZ* control according to unpaired t-test p value: < 0.05.

To further investigate possible involvement of flavins in the observed ampicillin treatment induced cellular AF, we used reporters to measure expression of *ribA*, *ribB*, *ribC* and *ribE*, which are essential genes required for FMN and FAD biosynthesis^29^ (Fig. S5). Expression of *ribA*, *ribC* and *ribE* genes increased significantly in cells treated with ≥1× MIC of ampicillin relative to untreated controls (Fig. 7a). The expression of the *ribB* gene increased significantly in cells treated with ≥4× MIC of ampicillin relative to untreated controls.

Finally, we tested the impact of the deletion of the *yeeO* gene that codes for the MATE transporter protein, which exports both FMN and FAD^30^ on ampicillin-induced AF (Fig. 7b). At 1× MIC of ampicillin, we found that the Δ*yeeO* strain had higher AF than the control strain, while there was no difference at 0.25× or 0.5× MIC. Taken together, our data suggest that flavins are major contributors to the observed increase in AF in ampicillin-treated cells.

### Protective induction of genes coding for flavoproteins in ampicillin-treated cells

It was estimated that 1 to 3% of all bacterial enzymes, most of which are involved in redox processes, depend on the FMN or FAD cofactors for their activity^14^. Many flavoproteins are involved in the response to various cellular damages, which may explain why flavin biosynthesis pathway is induced by cytotoxic treatments. Binding to flavin cofactors was experimentally confirmed for only a small number of proteins^31^. We examined the activity of promoters of 28 genes coding for the confirmed flavoproteins that we found in the Uri Alon library of transcriptional reporters^23^. In addition, we tested the promoter of the *soxR* gene which codes for the non-flavin-requiring protein that controls SoxRS regulon, including *ribA* gene^32^. This regulon is involved in the protection against superoxide and nitric oxide-induced stress. Finally, we used the *lacZ* gene promoter as a control. The advantage of this control reporter is that it can be fully induced with IPTG. All tested promoters were fused to the gene coding for GFP protein^23^. The cells were grown with and without ampicillin in 96-well microplates, and optical density and GFP fluorescence were measured for 3 hours (Table 1).

**Table 1:** Expression of 28 genes coding for flavoproteins, the *soxR* and *lacZ* genes in ampicillin-treated *E. coli* cells.

	Ampi 1/Ampi 0			Ampi 2 (MIC)/Ampi 0			Ampi 4/Ampi 0		
	Mean fold increase	Standard error	p value	Mean fold increase	Standard error	p-value	Mean fold increase	Standard error	p-value
**Controls**									
*lacZ*	0.97	0.06	**reference**	1.19	0.08	**reference**	3.25	0.23	**reference**
lacZ IPTG	0.98	0.01	0.9694	1.23	0.03	0.9674	2.68	0.11	0.7101
**Protection against ROS and reactive nitrogen species (RNS)**									
*soxR*	0.97	0.15	0.747	2.36	0.41	**0.0062**	11.29	1.46	**0.0003**
*fpr*	0.92	0.06	0.7568	2.14	0.13	**0.0035**	7.43	0.58	**0.0158**
*nfnB*	1	0.08	0.7189	2.29	0.31	**0.0024**	10.93	0.51	<**0.0001**
*norV/norW*	1.23	0.1	0.0908	3.77	1.04	**0.0003**	14.91	1.42	<**0.0001**
**Genes coding for the respiratory chain components**									
*poxB*	0.86	0.22	0.4071	2.23	0.28	**0.006**	8.21	1.5	**0.0096**
*ndh*	1.02	0.06	0.4608	1.86	0.09	**0.0368**	10.66	0.78	**0.0001**
*nuoA/nuoF*	1.38	0.13	**0.0192**	3.29	0.64	**0.0006**	10.9	1.61	**0.0006**
*ycaK*	1.26	0.08	**0.0221**	2.41	0.35	**0.0044**	8.9	0.36	**0.0029**
**Genes coding for proteins involved in different metabolic pathways**									
*cysJ*	1.92	0.73	**0.0499**	3.78	0.19	<**0.0001**	17.01	2.42	<**0.0001**
*ssuD/ssuE*	1.15	0.27	0.0704	5.96	1.18	<**0.0001**	16.33	2.01	<**0.0001**
*aroC*	1.14	0.3	0.2625	2.91	0.39	**0.0004**	19.1	2.43	<**0.0001**
*ilvI*	1.59	0.18	**0.006**	3.04	0.47	**0.0003**	11.07	0.94	**0.0002**
*metF*	0.77	0.05	0.1393	1.89	0.33	**0.0443**	7.02	1.12	**0.0347**
*dfp*	1	0.08	0.768	2.1	0.29	**0.0088**	9.55	0.58	**0.0007**
*mqo*	1.23	0.1	0.0699	3.15	0.38	<**0.0001**	9.76	1.95	**0.0024**
*wrbA-1*	1.11	0.04	0.0998	1.99	0.07	**0.01**	4.96	0.66	0.3019
*wrbA-2*	1.1	0.03	0.1287	1.78	0.06	0.0689	4.37	0.28	0.4863
*wrbA-3*	1.04	0.03	0.3303	2.3	0.09	**0.0009**	5.17	0.7	0.2311
*frdA*	1.18	0.15	0.2568	2.13	0.46	**0.0062**	6.12	0.86	0.1119
*ycdH/ycdM*	1.04	0.08	0.5753	2.78	0.42	**0.0005**	9.43	1.04	**0.0017**
**Not induced**									
*fldA*	1.01	0.02	0.6475	1.64	0.07	0.1935	7.74	0.39	**0.0086**
*gcl*	0.79	0.04	0.1099	0.77	0.08	0.5638	7.31	0.75	**0.018**
*glpA*	1.07	0.01	0.2036	1.59	0.04	0.2704	5.02	0.34	0.2777
*glpD*	1	0.01	0.7167	1.76	0.09	0.0802	4.19	0.24	0.5631
*lpd*	0.95	0.05	0.8387	1.61	0.06	0.2186	6.3	0.11	0.0683
*pdxH*	1.01	0.03	0.6753	1.5	0.04	0.4295	5.26	0.22	0.2582
*sdhA/sdhC*	0.85	0.03	0.2015	1.31	0.07	0.7818	3.4	0.32	0.9274
*uxaC*	1.08	0.05	0.2231	1.7	0.07	0.1295	6.34	0.35	0.072
*yieF/yieE*	0.76	0.03	0.0772	0.96	0.08	0.7004	2.81	0.29	0.777

Promoters of the tested genes were fused to the gene coding for the fast-folding green fluorescent protein (GFP; λ_ex_ 488/9/λ_em_ 525/20 nm). *E. coli* MG1655 strains (MIC ampicillin = 2 mg/L) carrying plasmids with these transcription reporter fusions were grown with a range of ampicillin concentrations. After 3 hours of incubation, GFP fluorescence (λ_ex_ 488/9/λ_em_ 525/20 nm) and OD_600nm_ of treated cells and non-treated control were measured using a fluorimeter. Fluorescence was normalized by the OD_600nm_. Each value represents the mean (+/−standard error) of the fluorescence increase compared with the non-treated condition of each strain from six independent experiments. Significant differences with the *lacZ* control according to the 1-way ANOVA Dunn’s test < 0.05 are presented in bold.

We broadly classified the gene promoters that showed significant induction when compared to the *lacZ* control, in three functional categories according to the gene functions described in the EcoCyc *E. coli* database (http://ecocyc.org/). (i) Genes coding for protection against ROS and reactive nitrogen species (RNS); *soxR*, sensor and transcriptional regulator of the SoxRS regulon; *fpr*, a member of the SoxRS regulon, which is involved in the protection against oxidative agents such as methyl viologen and paraquat; *nfnB*, reduction of nitroaromatic compounds including the antibiotics nitrofurazone and nitrofurantoin; *norV*/*norW*, nitric oxide reductase. (ii) Genes coding for the respiratory chain components: *poxB*, *ndh*, *nuoA* and *nuoF*. (iii) Genes coding for proteins involved in different metabolic pathways: Sulfur metabolism: *cysJ*, sulfite reductase involved in the assimilation of sulfate (A *cysJ* mutant is less sensitive to paraquat than wild type); *ssuD/ssuE*, alkanesulfonate monooxygenase allowing utilization of alkanesulfonates as a sulfur source (*ssuD* deletion mutant is sensitive to heat shock). Amino acid metabolism: *aroC* involved in biosynthesis of aromatic amino acids; *ilvI*, participates both in valine and isoleucine biosynthesis; *metF*, involved in biosynthesis of methionine. Energy metabolism: *dfp*; catalyzes two sequential reactions in the coenzyme A biosynthetic pathway; *mqo*, TCA cycle enzyme malate:quinone oxidoreductase; *wrbA* having an NAD(P)H:quinone oxidoreductase activity; *frdA*, catalytic subunits of the fumarate reductase complex.

**Figure 8:**
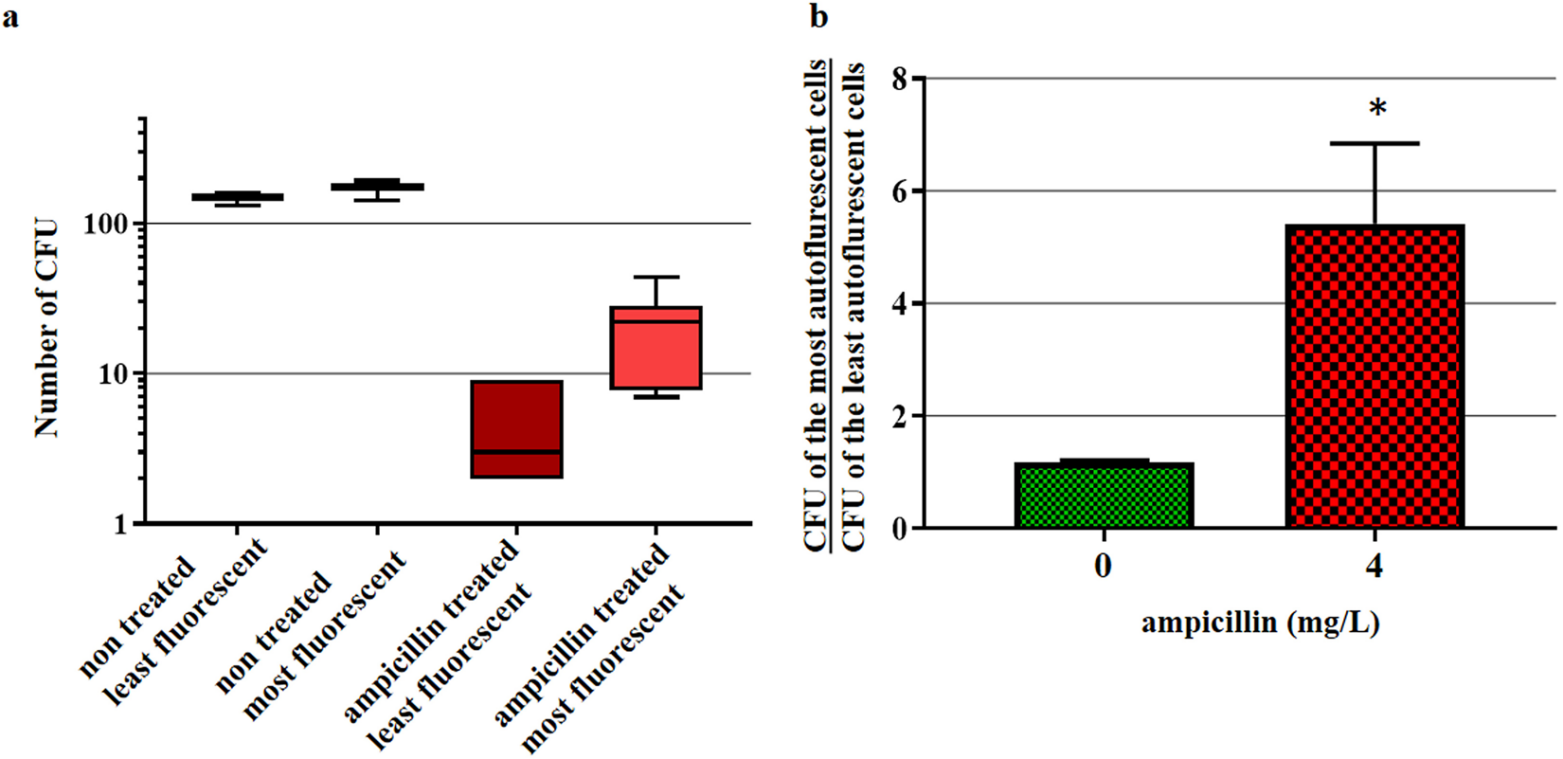
Autofluorescence as a predictor of the survival of ampicillin-treated *E. coli* cells. *E. coli* 7705035 cells were incubated with 4 mg/L ampicillin (MIC). After 2 hours of incubation, the autofluorescence of treated and non-treated control cells was analyzed using the S3e cell sorter. 200 of the least and 200 of the most autofluorescent cells were sorted and plated on LB agar. After overnight growth, colony-forming units (CFU) were counted to assess the bacterial survival. (**a**) Box plot of the CFUs. (**b**) The mean ratio (+/−standard error) between the number of CFUs of the most and the least fluorescent cells of treated and non-treated control cells per experiment. Presented data are from six independent experiments. The asterisk represents significant difference according to paired T-test p value: 0.034.

Increased transcription of genes coding for respiration, energy metabolism, different biosynthetic pathways and protection against ROS and RNS indicates that cells treated with ampicillin induce different cellular mechanisms in order to fix treatment-induced cellular damage and survive. If the increase in these flavoproteins is directly correlated with the increased AF, then the increase in AF should correlate with higher cellular robustness. In order to test this hypothesis, we incubated *E. coli* cells with the 1× MIC of ampicillin for 2 hours, after which 200 cells per condition, with low and high AF intensity were sorted using FACS, plated on LB agar plates without antibiotic, incubated overnight at 37 °C and compared with untreated controls (Fig. 8). Untreated bacteria did not show any significant difference in the plating efficiency between low AF and high AF populations. However, regarding ampicillin-treated bacteria, the plating efficiency of the high AF population was about 5-fold higher than that of the low AF population. This result supports our hypothesis that AF increase reveals the induction of protective responses to severe stresses.

## Discussion

In this study, we examined how the AF of *E. coli* cells changes as a function of exposure to the different stressors. We found that bactericidal stressors, such as ß-lactam ampicillin, significantly increased cellular AF and that *de novo* protein synthesis is required for the AF increase. We also showed that an increased AF is not an artifactual consequence of an antibiotic-induced cellular morphology. Several lines of evidence suggest that most of the observed increase in the AF comes from flavins. Firstly, the excitation and emission spectra of the AF of ampicillin-treated cells correspond to the spectra of purified flavins: riboflavin, FMN and FAD. Similarly, Mihalcescu *et al*.^11^ have studied AF of *E. coli* grown in comparable conditions and have shown that flavins FMN, FAD and riboflavin, were responsible for more than 80% of the fluorescence at wavelength 525 nm. They also reported that most of the remaining 20% of fluorescent molecules could be attributed to other flavin derivatives. Secondly, *ribA*, *ribB*, *ribC*, and *ribE* genes required for the FMN and FAD biosynthesis are induced with the ampicillin treatment. Thirdly, ampicillin-treated cells with deletion of the *yeeO* gene that codes for the MATE transporter protein exporting both FMN and FAD, have higher AF increases than ampicillin-treated cells with the functional *yeeO*. Fourthly, many of the tested genes coding for flavoproteins are overexpressed in ampicillin-treated cells.

We also demonstrated that increased AF is not just a collateral consequence of the cytotoxic treatment, but that it corresponds to the activation of a complex adaptive response that increases cell robustness to severe stresses. This is primarily illustrated by the fact that the plating efficiency of the high AF intensity ampicillin-treated cells was, on average, 5-fold higher than that of the low AF intensity ampicillin-treated cells. Cellular functions of the observed ampicillin-induced genes indicate how increased flavin production can contribute to increased cell robustness. These genes code for flavoproteins that are involved in respiration, energy metabolism, different biosynthetic pathways and protection against ROS and RNS. The observed induction of the *ribA*, *ribB*, *ribC* and *ribE* genes by ampicillin treatment is important for increased supply of the FMN or FAD cofactors for flavoproteins. In addition, the *ribA* gene is a member of the SoxRS regulon and is also inducible by paraquat and superoxide-generating compounds^32^. This protein can also hydrolyze 8-oxo-dGTP which is a potent mutagenic substrate for DNA synthesis^33^. Importantly, AF increased significantly relative to the untreated control when the concentration of bactericidal stressor was close to or above the MIC. Therefore, AF can be used for the evaluation of susceptibility of bacterial pathogens to the bactericidal, non-protein synthesis inhibiting antibiotics.

A common reaction of the prokaryotic and eukaryotic cells to stress is the increase in energy production, which is required to deal with macromolecular damages. For this, cells increase respiratory activity, which increases ATP production, but also accidental ROS production that must be rapidly eliminated. The AF of the NAD and FAD cofactors is widely used to follow changes in energy production by the respiratory chain in eukaryotic cells, as well as to monitor the redox state of the stressed cells^34,35^. FAD fluorescence can be quenched when they are protein bound. However, it was also observed that FAD fluorescence increases when they are bound to mitochondrial lipoamide dehydrogenase (LipDH) and electron transfer flavoprotein (ETF), which are responsible for 75% of the flavin autofluorescence in rat liver mitochondria^36,37^. Importantly, it was reported that the amount of electron-transfer fluorescent flavoproteins correlates with respiration rates in eukaryotic cells^34,36,38^.

NAD and FAD fluorescence reflects the redox state of cells. For example, the reduced form of the NAD, NADH, is strongly fluorescent, while its oxidized form NAD^+^ is poorly fluorescent^2,4^. Thus, it was observed that oxidative stress results in a decrease in fluorescence due to the oxidation of NADH in human cells treated with hydrogen peroxide or menadione^39,40^. The opposite is the case for FAD, which is fluorescent in the oxidized form and not in the reduced form. Our data show that the spectra of ampicillin-treated cells are very similar to the spectra of oxidized FAD, while they did not fit with the spectra of reduced FAD^41^.

An increase in the oxidized/reduced ratio of flavins may result in increased fluorescence without necessarily increasing flavin concentration. This could be the case in the cells treated with sodium hypochlorite, which is a potent oxidizing agent. However, the neosynthesis of flavoproteins also contributes to an increase in AF in stressed cells. The flavoproteins play a crucial role in energy metabolism, drug and xenobiotic metabolism, immune defense, chromatin remodeling, DNA repair, and apoptosis^14,42,43^ (and references therein). Therefore, the increased AF of the eukaryotic cells corresponds to the induction of responses to cope with the deleterious effects of the life-threatening stress, like we observed in bacterial cells. For example, increased flavin concentrations were reported to be involved in the increased AF of the human and murine cells following ionizing radiation^44^.

In conclusion, this study shows that increased AF indicates that prokaryotic and eukaryotic cells are experiencing severe life-threatening stress. The observed increase in AF may come from both oxidation and synthesis of flavins. The relative contribution of oxidation and/or flavin neosynthesis to the AF increase is likely determined by the nature and strength of the stressor. Therefore, measurement of AF variations in general and that of flavins and flavoproteins in particular, can be used to study the cellular metabolic state and the molecular mechanisms involved in the response to stress, but also for various diagnostic applications in prokaryotic and eukaryotic cells.

## Material and Methods

### Strains, growth media and culture conditions

Bacterial and yeast strains, as well as human cell lines used in this study, are described in Table S1. Overnight liquid bacterial cultures were grown aerobically in 50 mL Falcon tubes in Lysogeny broth (LB) medium, Difco LB broth, Miller (Becton Dickinson, Franklin Lakes, USA) at 37 °C with shaking. For experiments, overnight cultures were diluted 10^4^-fold in appropriate media supplemented with antibiotics or chemicals when needed, and grown aerobically at 37 °C with shaking in 50 mL Falcon tubes.

Yeast strains were grown aerobically in 50 mL Falcon tubes at 30 °C for 48 hours in liquid Yeast Extract-Peptone-Dextrose (YPD) (Becton Dickinson) medium supplemented with 2% (final) glucose. For experiments, 48 hour yeast cultures were diluted 10^4^-fold in liquid YPD medium and grown aerobically at 30 °C with shaking.

HeLa cells were cultured in Dulbecco’s Modified Eagle’s medium (DMEM) (Thermo - Fisher Scientific, Waltham, USA) supplemented with 10% fetal bovine serum (Gibco sera, South American origin). The cells were grown in a humidified atmosphere of 5% CO_2_ at 37 °C and the medium was changed every 2 days.

### Antibiotics and chemicals

Ampicillin, ciprofloxacin, gentamicin, imipenem, meropenem and tetracycline were purchased from Sigma-Aldrich (Saint Louis, USA). 100× stock solutions of each antibiotic were prepared in water and then diluted in culture media to appropriate concentrations. Alexa Fluor™ 633 Hydrazide (AF633H), TOPRO^©^-3 and 16% Paraformaldehyde (PFA) methanol free were purchased from Thermo - Fischer Scientific and diluted in Phosphate Buffer Saline (PBS) (Gibco, Thermo - Fischer Scientific). Flavin Mono Nucleotide (FMN), Flavin Adenine Dinucleotide (FAD) and Riboflavin were purchased from Sigma-Aldrich.

### Flow cytometry and cell sorting

We used a Gallios (Beckman Coulter, Brea, USA) analyzer 10 color, 4 laser system to analyze samples. FL1 channel (excitation at 488 nm with bandpass filters of 525/20 nm) was used for autofluorescence analysis. FL6 channel (excitation at 638 nm with bandpass filters of 660/20 nm) was used for AF633H and TOPRO^®^ - 3 analysis. Samples were run at a rate between 1,000 and 2,000 events/s; 20,000 to 50,000 events were collected depending on sample type (prokaryotic or eukaryotic cells). Data acquisition was performed using the Kaluza software.

For ImageStream cytometry, samples were acquired on the ImageStreamX MarkII system (Amnis Corporation, Seattle, USA) using a 488 nm laser (Bandpass filter 505–560 nm). 20,000 events per condition were collected. The IDEAS software (Amnis) was used to analyze the measurements. Image acquisition and image analysis were performed using the IMAG’IC Facility (Institut Cochin, Paris, France).

For cell sorting, we used a S3e cell sorter (Bio-Rad, Hercules, USA). FL1 channel (excitation at 488 nm emission at 525/30 nm). Samples were run at an event rate of 1,200 events/s and 200 cells per condition were sorted in 200 µL 1x PBS with the “single cell” sort mode. Data acquisition was performed using the ProSort^TM^ software (Bio-Rad).

### Autofluorescence variation analysis

Overnight bacterial and 48 hour yeast cultures were diluted in 20 mL LB or YPD medium respectively, to obtain a final 600 nm Optical Density (OD_600nm_) ≤ 0.05 with a Biophotometer plus (Eppendorf, Hamburg, Germany). Cultures were incubated aerobically at 37 °C for bacteria and 30 °C for yeast, in 50 mL Falcon tubes with orbital shaking until reaching an OD_600nm_ of 0.2 (AU). At this point, the bacterial cultures were subdivided into 50 mL Falcon tubes and sodium hypochlorite or different amounts of antibiotics were added to obtain a range of antibiotic concentrations. Incubation was performed at 37 °C for bacteria and 30 °C for yeast, with agitation. Samples of treated and untreated control cells were taken every hour, diluted in 500 µL 1x PBS to obtain around 1,000 to 2,000 events/s for bacteria and 500 to 1,000 events/s for yeast and immediately analyzed with the flow cytometer. For the Imagestream X analysis, aliquots of cells were fixed with 2% (final) methanol free paraformaldehyde for 10 minutes at room temperature, washed with PBS and kept on ice until analysis.

HeLa cells were treated with sodium hypochlorite (0.08%; 0.16%, 0.32%). After 1 hour of incubation, cells were collected, washed with PBS, and analyzed by flow cytometry.

### Cell viability assessment

Samples of bacterial cells prepared for autofluorescence variation analysis (see above) were killed by incubation at 65 °C for 30 min, and stained with 2 µg/mL AF633H or 0.5 µM TOPRO^®^-3 iodide dyes. Cells labeled with AF633H were incubated for 15 min on ice and in the dark, and then washed with cold PBS before flow cytometry analysis. Cells labeled with TOPRO^®^-3 were incubated at room temperature for 15 min in the dark before flow cytometry analysis.

### Analysis of the fluorescence spectra

Bacterial overnight cultures were diluted in LB or M9 - glucose medium (Becton Dickinson) (supplemented with 0.003% B1 vitamin and 1 mM MgSO_4_) to obtain an OD_600nm_ ≤ 0.05. M9 medium was used to avoid background fluorescence from proteins and other natural molecules usually found in complex media like LB. Diluted cultures were subsequently incubated aerobically at 37 °C in 50 mL Falcon tubes with orbital shaking until reaching an OD_600nm_ of 0.2 (AU). At this point, bacterial cultures or medium without bacteria as a control, were distributed in 5 Falcon tubes and different amounts of antibiotic were added to each tube to obtain a range of antibiotic concentrations. Tubes were subsequently incubated at 37 °C with agitation. Every hour, 100 µL of the bacterial cultures or the control medium were sampled and added to the wells of the 96-well black microplate containing 100 µL 1x PBS.

Purified flavonoid compounds were diluted in sterile water to obtain a stock concentration of 20 µM. 100 µL of the stock solution were added to 100 µL 1x PBS in the 96-well microplate. OD_600nm_ measurements and fluorescence scans were performed using an Infinite M200 PRO and an Infinite M1000 (Tecan, Männedorf, Switzerland) reader.

### Gene expression measurements

To evaluate how antibiotic treatments affect gene expression, we used strains from a library of *E. coli* strains carrying transcriptional fusions of gfp to different promoters^23^. Overnight cultures containing 12.5 µg/mL kanamycin were diluted in LB medium to obtain an OD_600nm_ of 0.1. 200 µL samples were then dispensed in the 96-well microplates. After 2 hours of incubation at 37 °C with orbital shaking allowing to obtain bacteria in an exponential growth phase, 40 µL of culture were added to a 37 °C warmed microplate containing a range of ampicillin concentrations (0 to 16 mg/L). 25 µL of mineral oil were added to the top of each well to prevent evaporation. Using a programmable robotic system “Freedom Evo” (Tecan), plates were incubated at 37 °C. OD_600nm_ and GFP fluorescence (λ_ex_ 488/9 nm λ_em_ 525/20 nm) were measured using an Infinite M200 PRO. *lacZ* promoter was used as a negative control. When the same promoter was used as a positive control, 0.2 mM IPTG were added to the growth medium. The medium background was subtracted and GFP fluorescence was divided by the OD_600nm_. Gene expression was presented as the increase in fluorescence compared to the non-treated condition.

### Autofluorescence and cell survival experiment

Overnight *E. coli* cultures were diluted in 20 mL LB in 50 mL Falcon tubes to a final OD_600nm_ ≤ 0.05, and incubated aerobically at 37 °C with orbital shaking until an OD_600nm_ of 0.2 (AU) was reached. Half of the obtained bacterial cultures were treated with 4 mg/L of ampicillin, which corresponds to the MIC, while the other half was used as an untreated control. After 2 hours of incubation, aliquots of the untreated and treated cultures were diluted in PBS and analyzed using the S3e cell sorter. Samples were run at 1,200 events/s, the FSC/FL1 dot plot was established and 2 gates were defined. The first gate corresponded to the least fluorescent cells and the second to the most fluorescent ones. 400 cells in each gate were sorted into 200 µL 1X PBS using the “single cell” sort mode. A 100 µL suspension of each sorted cell population was deposited on LB agar plates without ampicillin allowing cell recovery. Colony forming units were counted after overnight incubation at 37 °C.

### Data analysis

Flow cytometry data analysis and fluorescence normalization were performed using the Flowjo^©^ V10.2 software (Flowjo, Ashland, USA). Data from the microplate reader and Imagestream X were processed using Magellan 7.2 (Tecan) and Excel^©^ 2010 (Microsoft^®^, Redmond, USA). Statistical analyses were performed using Prism V7.0 (GraphPad^TM^, La Jolla, USA). FCS Express V4 (*De novo* software, Los Angeles, USA) was used to export raw data from the flow cytometer for correlation analysis.

## Electronic supplementary material

Supplementary dataset
