# Supplementary material for: Strong increase in the autofluorescence of cells signals struggle for survival
Source: Sci Rep. 2018 Aug 14;8:12088. doi: 10.1038/s41598-018-30623-2 (PMC6092379; doi:10.1038/s41598-018-30623-2)
Supplement: Supplementary file 1 — Supplementary dataset [file 41598_2018_30623_MOESM1_ESM.doc]

**Supplemental material**

**Strong increase in the autofluorescence of cells signals struggle for survival**

Jérémy Surre^1,2^, Claude Saint-Ruf^1,3^, Valérie Collin^2^, Sylvain Orenga^2^, Mahendrasingh Ramjeet^2^, and Ivan Matic^1,4,*^

^1^INSERM U1001, Université Paris Descartes, Sorbonne Paris Cité, Faculté de Médecine Paris Descartes, Paris, France.

^2^bioMérieux SA, Microbiology Unit, R&D Microbiology, La Balme les Grottes, France.

^3^ INSERM, U1016, CNRS, UMR8104,^3^ Institut Cochin, Paris, France.

^4^Centre National de la Recherche Scientifique (CNRS), 75016 Paris, France.

^*^Corresponding author : ivan.matic@inserm.fr

| 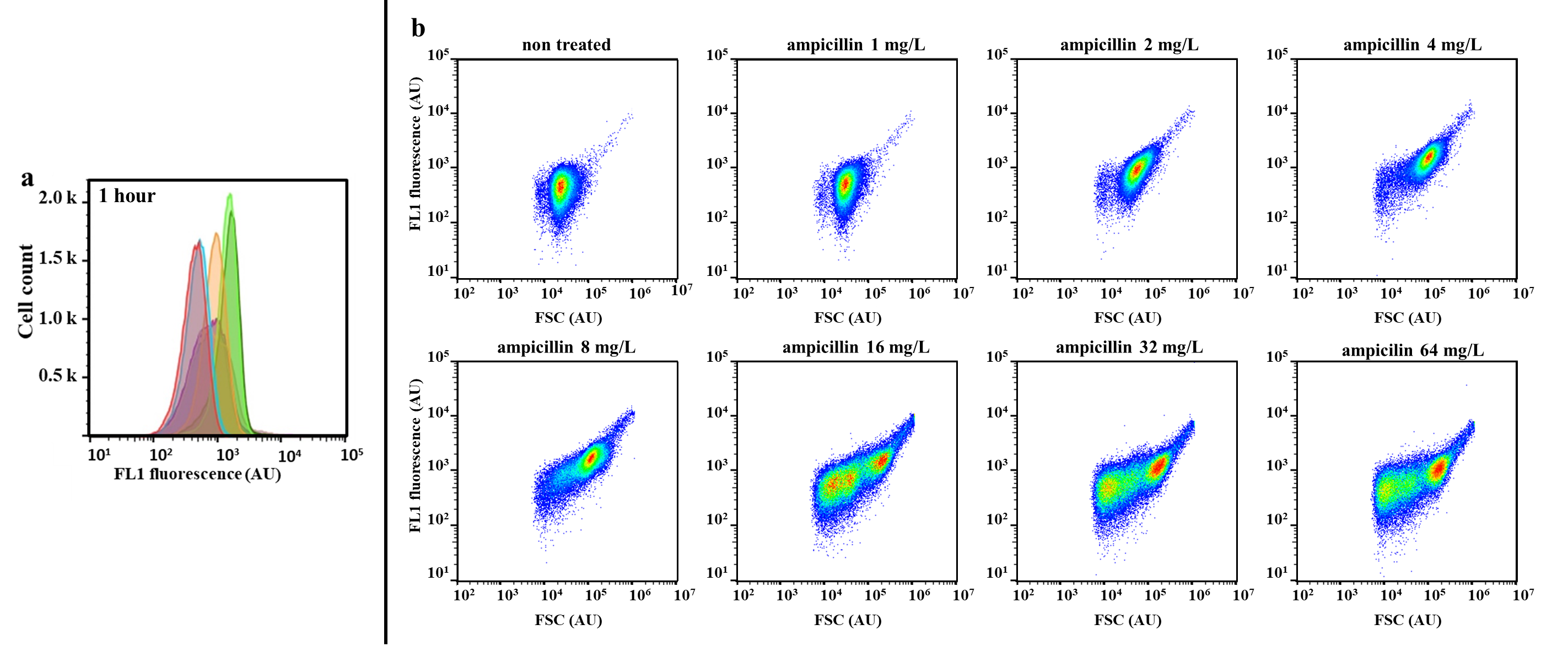 |
| --- |
| 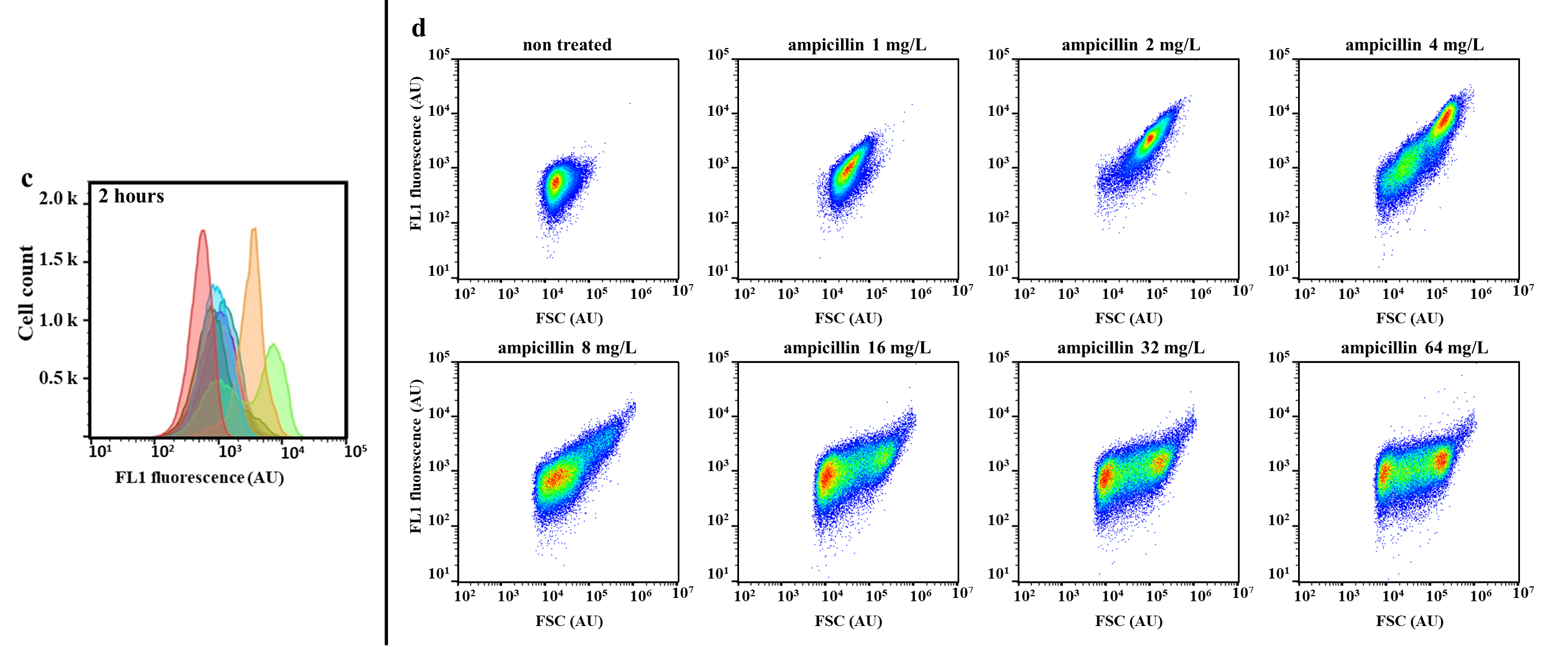 |
| 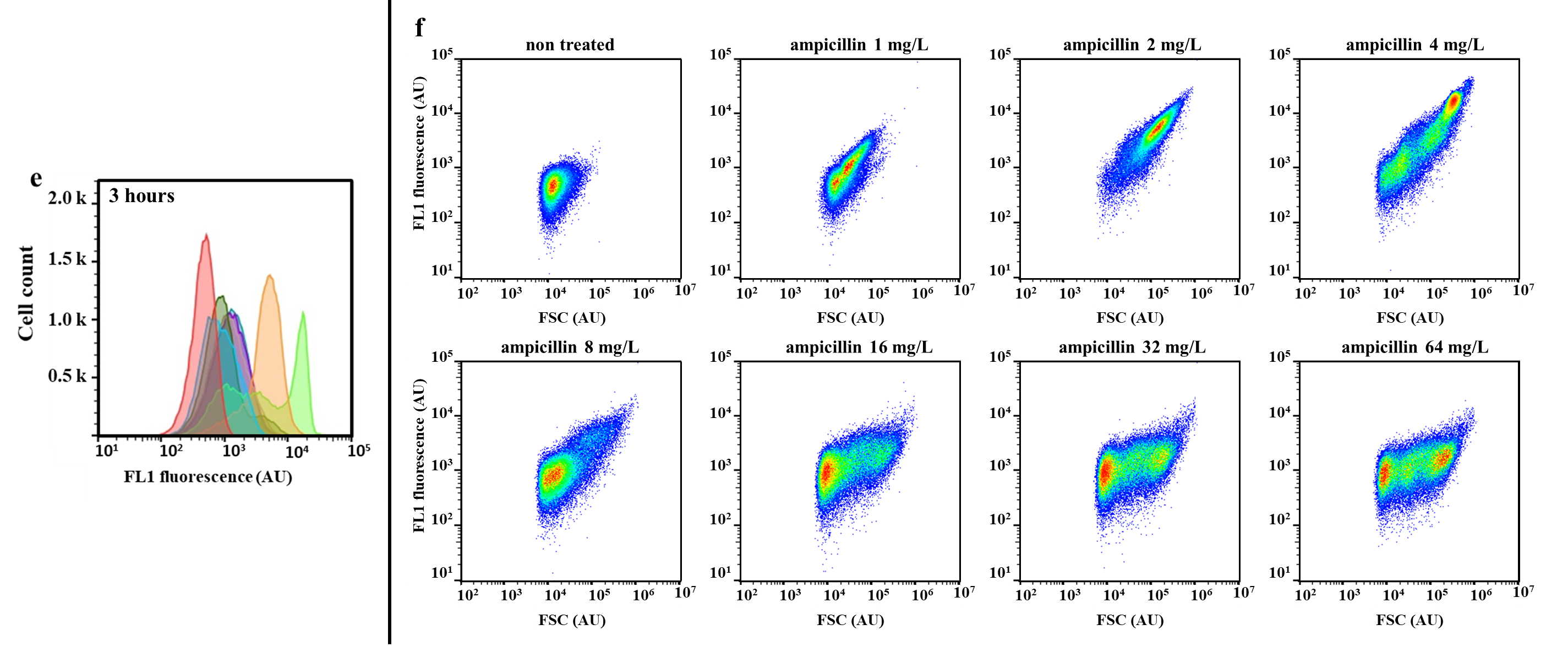 |

**Supplemental Figure S1 - panel 1: Effect of ampicillin treatment on the forward light scattering and autofluorescence intensity of *E. coli* cells.** Exponentially growing *E. coli* 7705035 cells (ampicillin MIC = 4 mg/L) were treated with a range of ampicillin concentrations for 3 hours. Autofluorescence and forward light scattering (FSC) of treated cells and untreated control were measured after 1, 2 and 3 hours of incubation using a flow cytometer. (**a**), (**c**), (**e**) The change in the distribution profiles of the cellular autofluorescence (λ_ex_ 488 nm / λ_em_ 525/20 nm) at 1, 2 and 3 hours respectively, for the ampicillin concentrations: 0 (red), 1 (light blue), 2 (orange), 4 (light green), 8 (dark green), 16 (light violet), 32 (dark violet) and 64 mg/L (dark blue). (**b**), (**d**), (**f**) Two-dimensional representation of the FSC and FL1 (λ_ex_ 488 nm / λ_em_ 525/20 nm) of each cell (n = 50,000) at 1, 2 and 3 hours of treatment respectively. The overlap of red, light blue, orange, light green and dark green produces a brown area. This panel presents results of one representative experiment, which was independently repeated three times.

| 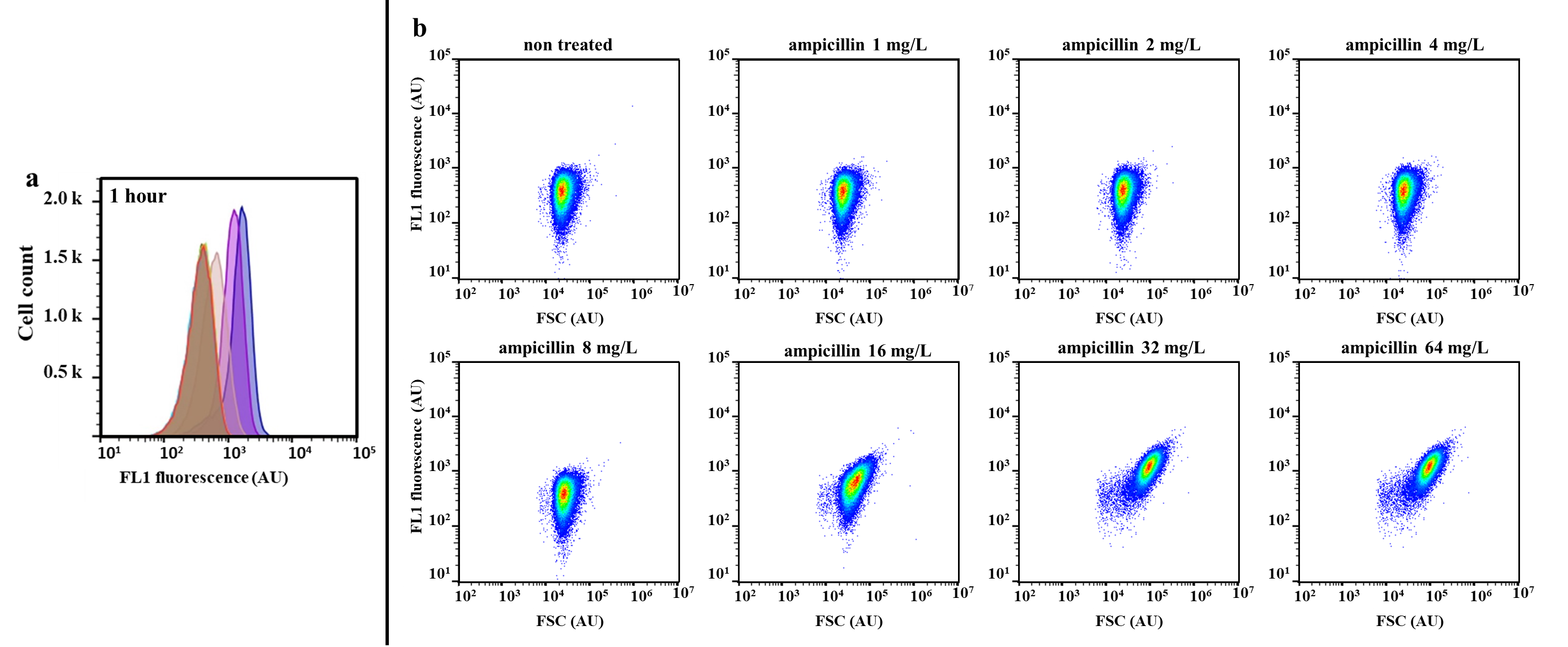 |
| --- |
| 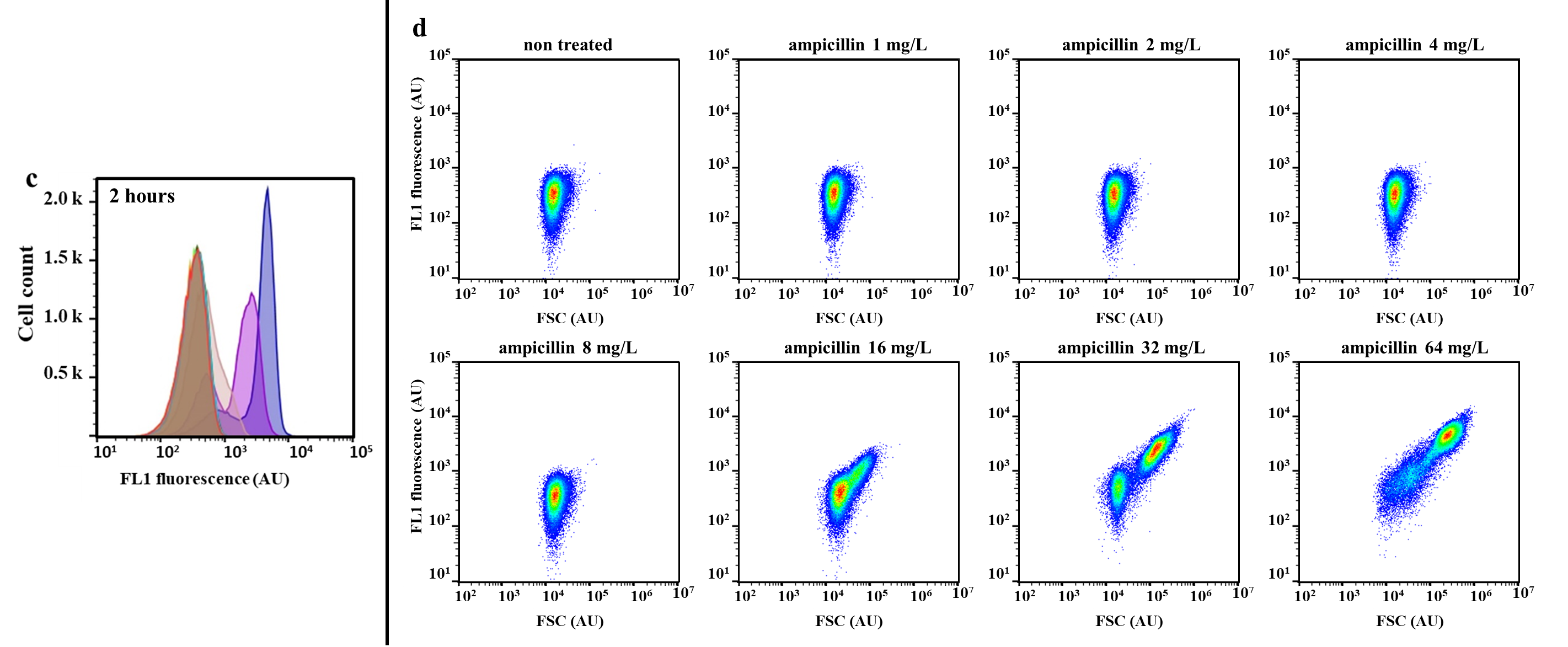 |
| 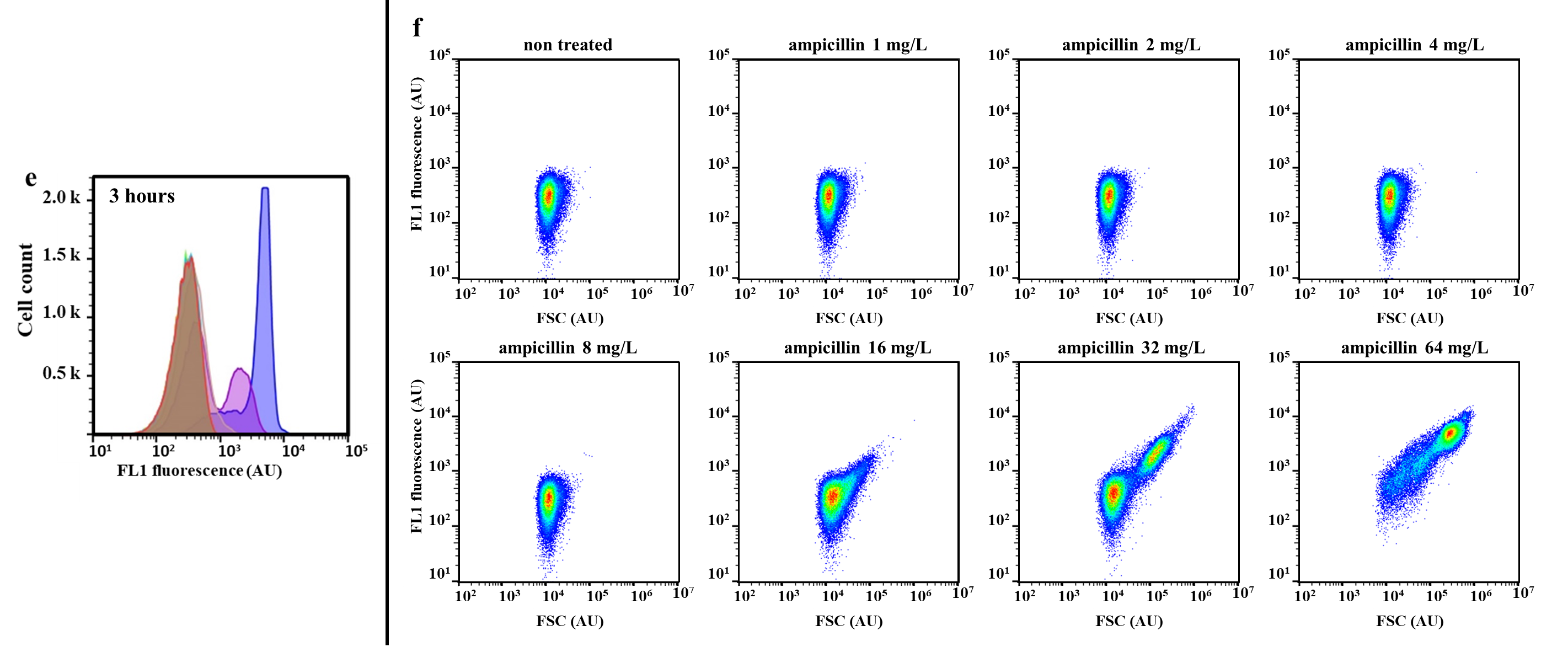 |

**Supplemental Figure S1 - panel 2: Effect of ampicillin treatment on the forward light scattering and autofluorescence intensity of *E. coli* cells.** Exponentially growing *E. coli* 8812112 cells (ampicillin MIC = 64 mg/L) were treated with a range of ampicillin concentrations for 3 hours. Autofluorescence and forward light scattering (FSC) of treated cells and untreated control were measured after 1, 2 and 3 hours of incubation using a flow cytometer. (**a**), (**c**), (**e**) The change in the distribution profiles of the cellular autofluorescence (λ_ex_ 488 nm / λ_em_ 525/20 nm) at 1, 2 and 3 hours respectively, for the ampicillin concentrations: 0 (red), 1 (light blue), 2 (orange), 4 (light green), 8 (dark green), 16 (light violet), 32 (dark violet) and 64 mg/L (dark blue). (**b**), (**d**), (**f**) Two-dimensional representation of the FSC and FL1 (λ_ex_ 488 nm / λ_em_ 525/20 nm) of each cell (n = 50,000) at 1, 2 and 3 hours of treatment respectively. The overlap of red, light blue, orange, light green and dark green produces a brown area. This panel presents results of one representative experiment, which was independently repeated three times

| 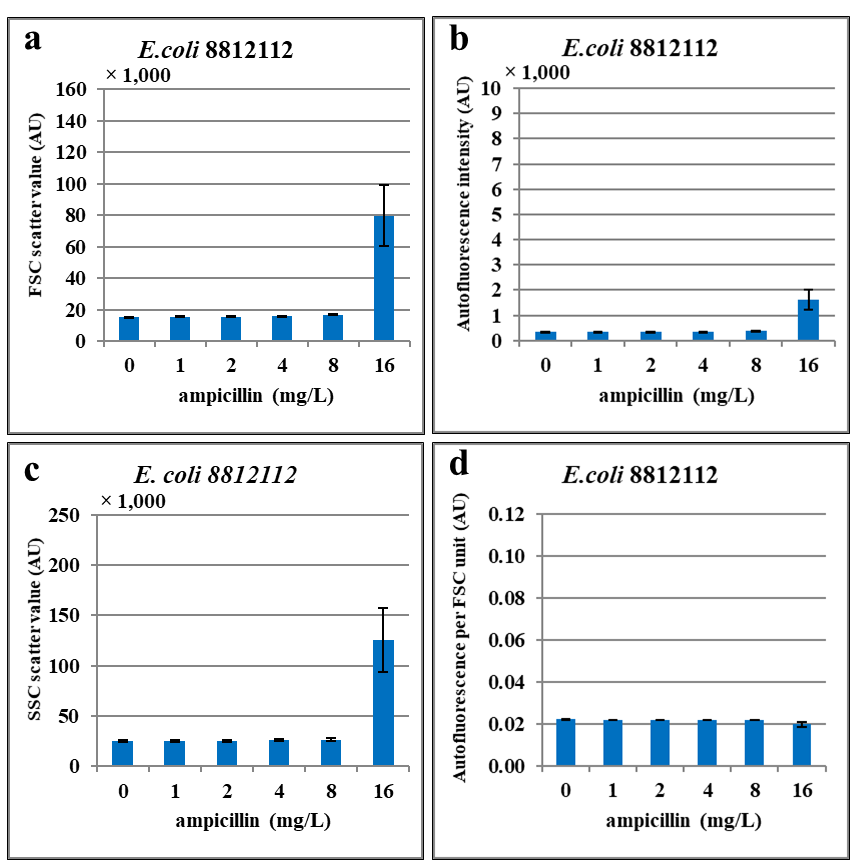 | 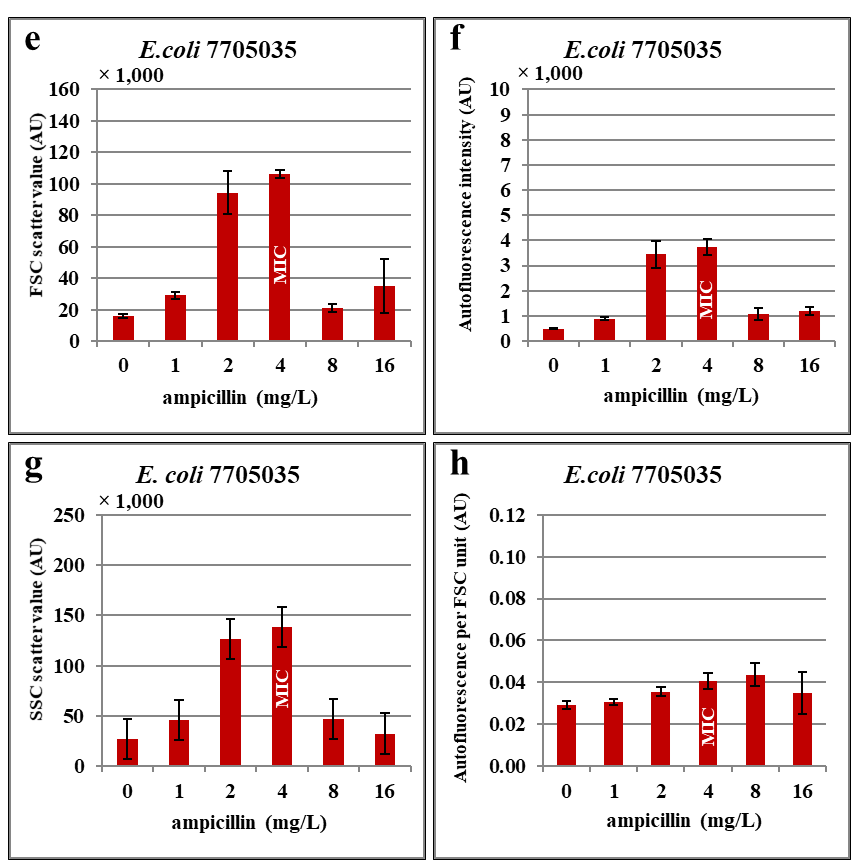 |
| --- | --- |
| 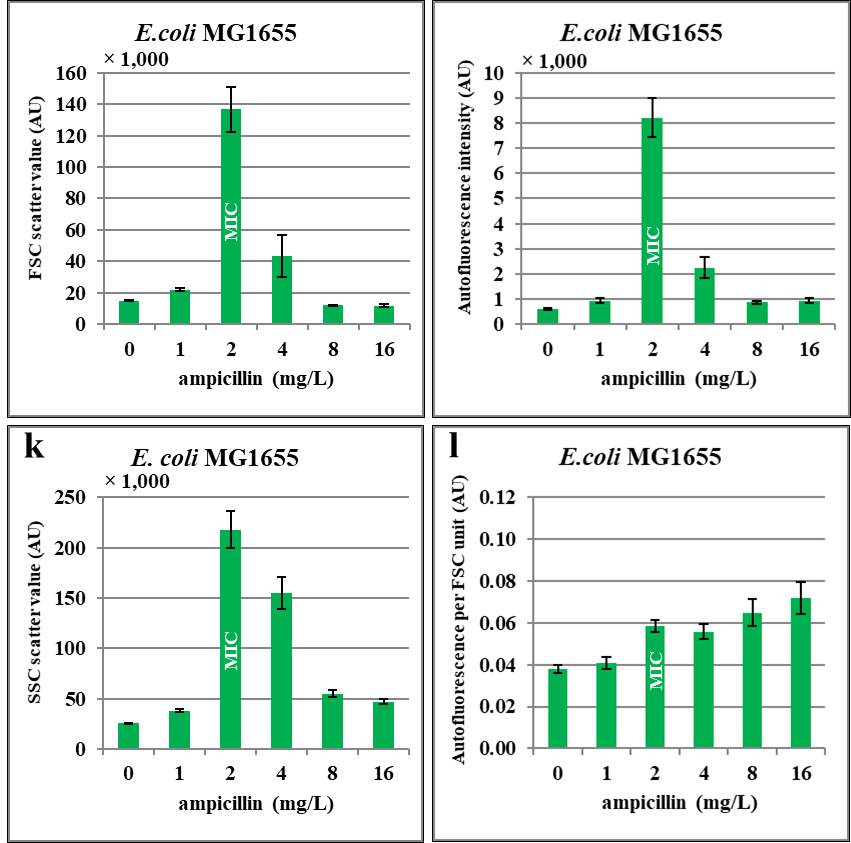 | 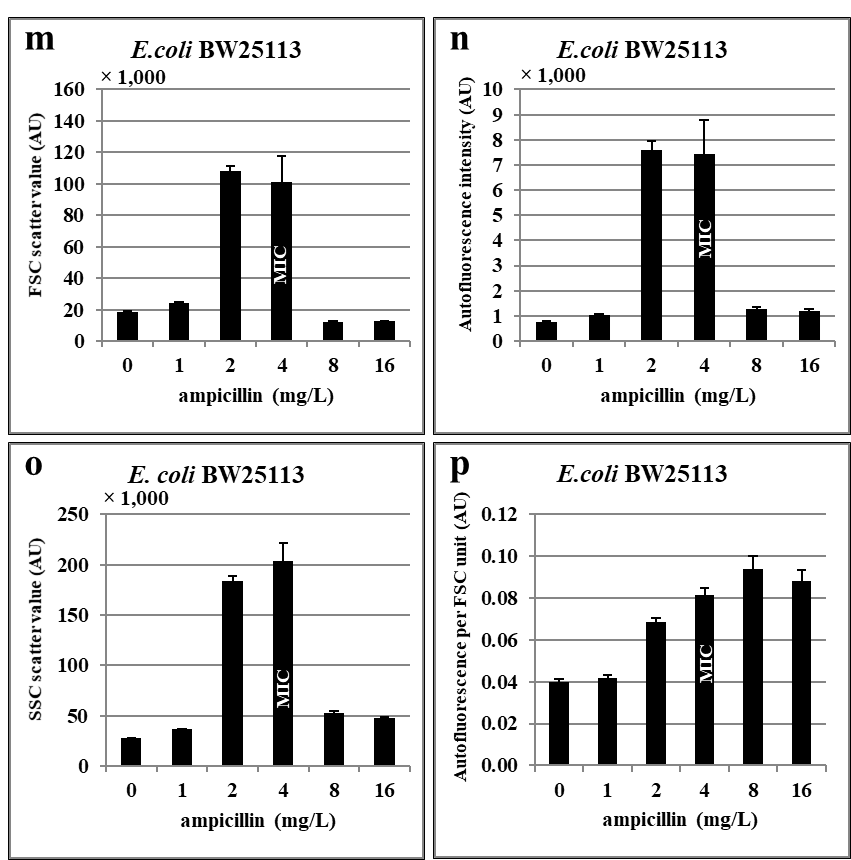 |

**Supplemental Figure S2, panel 1: Change in the FSC, autofluorescence, SSC and autofluorescence per FSC unit of *E. coli* cells after 2 hours of ampicillin treatment.**  Exponentially growing *E. coli* 8812112 (ampicillin MIC = 64 mg/L) (blue), *E. coli* 7705035 (ampicillin MIC = 4 mg/L) (red), *E. coli* MG1655 (ampicillin MIC = 2 mg/L) (green) and *E. coli* BW25113 (ampicillin MIC = 4 mg/L) (black) cells were treated with a range of ampicillin concentrations. After 2 hours of treatment, 50,000 cells were collected using a flow cytometer. (**a, e, i, m**) FSC value (**b, f, j, n**) Autofluorescence intensity (λ_ex_ 488 nm / λ_em_ 525/20 nm). (**c, g, k, o**) SSC value and (**d, h, I, p**) autofluorescence intensity per FSC unit. Each value represents the mean +/- standard error of the median of the cell populations after 2 hours of at least three independent experiments.

| 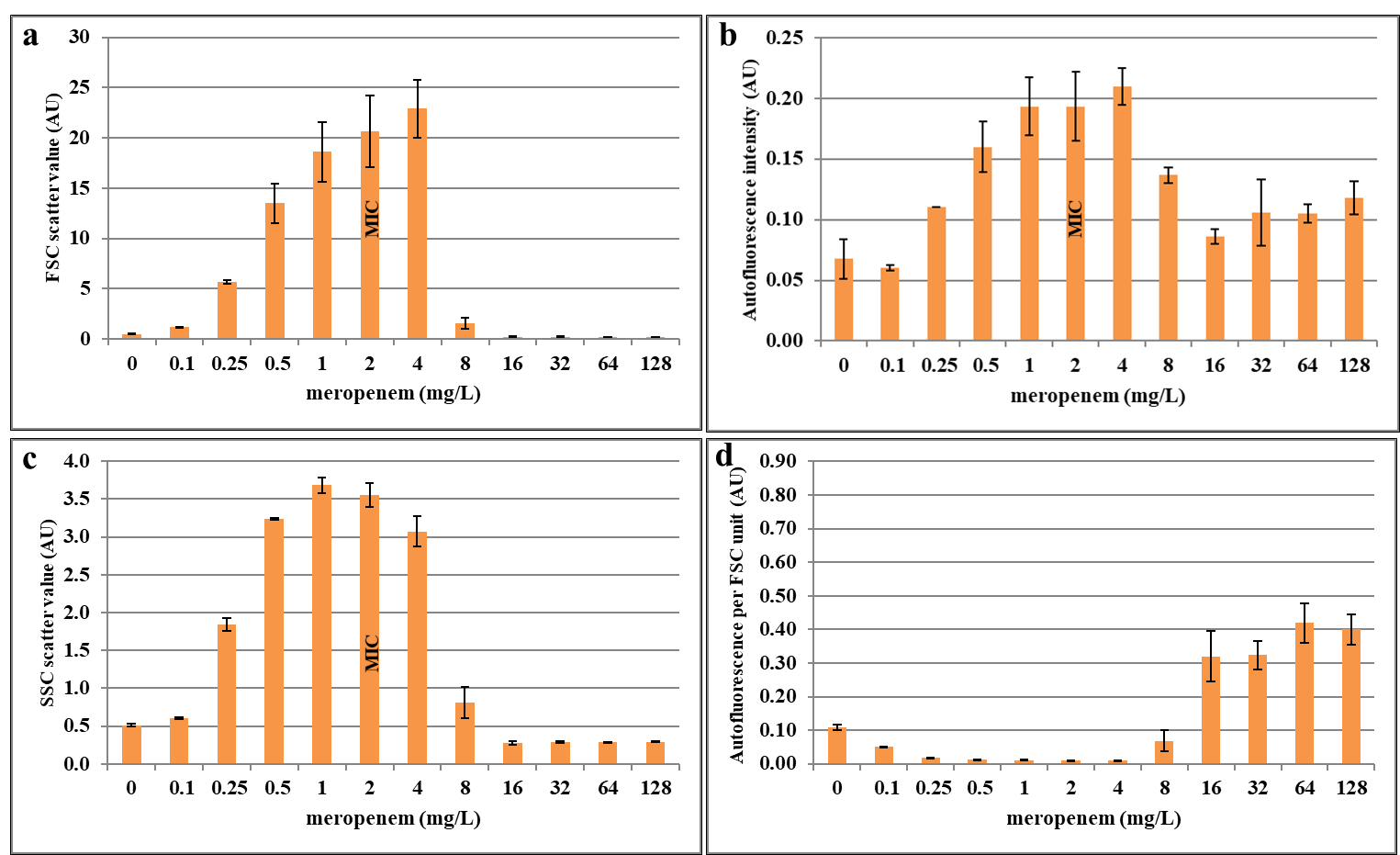 |
| --- |
| 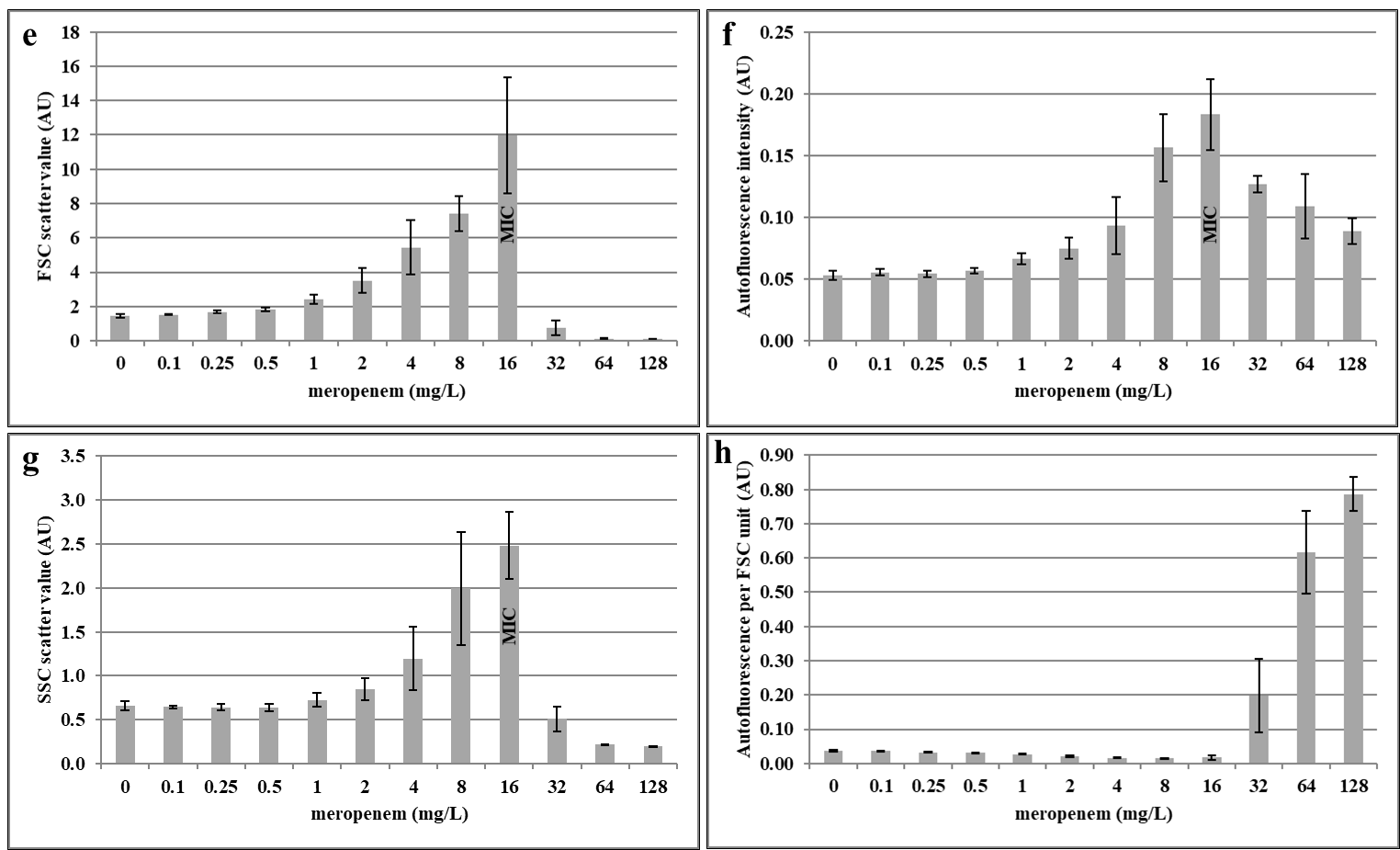 |

**Supplemental Figure S2, panel 2: Change in the FSC, autofluorescence, SSC and autofluorescence per FSC unit of *S. marcescens* and *K. pneumoniae* cells after 2 hours of meropenem treatment.** Exponentially growing *S. marcescens* (orange) cells (meropenem MIC = 2 mg/L) and *K. pneumoniae* (grey) cells (meropenem MIC = 16 mg/L) were treated with a range of meropenem concentrations. After 2 hours of treatment, 50,000 cells were collected using a flow cytometer. (**a, e**) FSC value (**b, f**) Autofluorescence intensity (λ_ex_ 488 nm / λ_em_ 525/20 nm). (**c, g**) SSC value. (**d, h**) Autofluorescence intensity per FSC unit. Each value represents the mean +/- standard error of the median of the cell populations after 2 hours of at least three independent experiments.

| 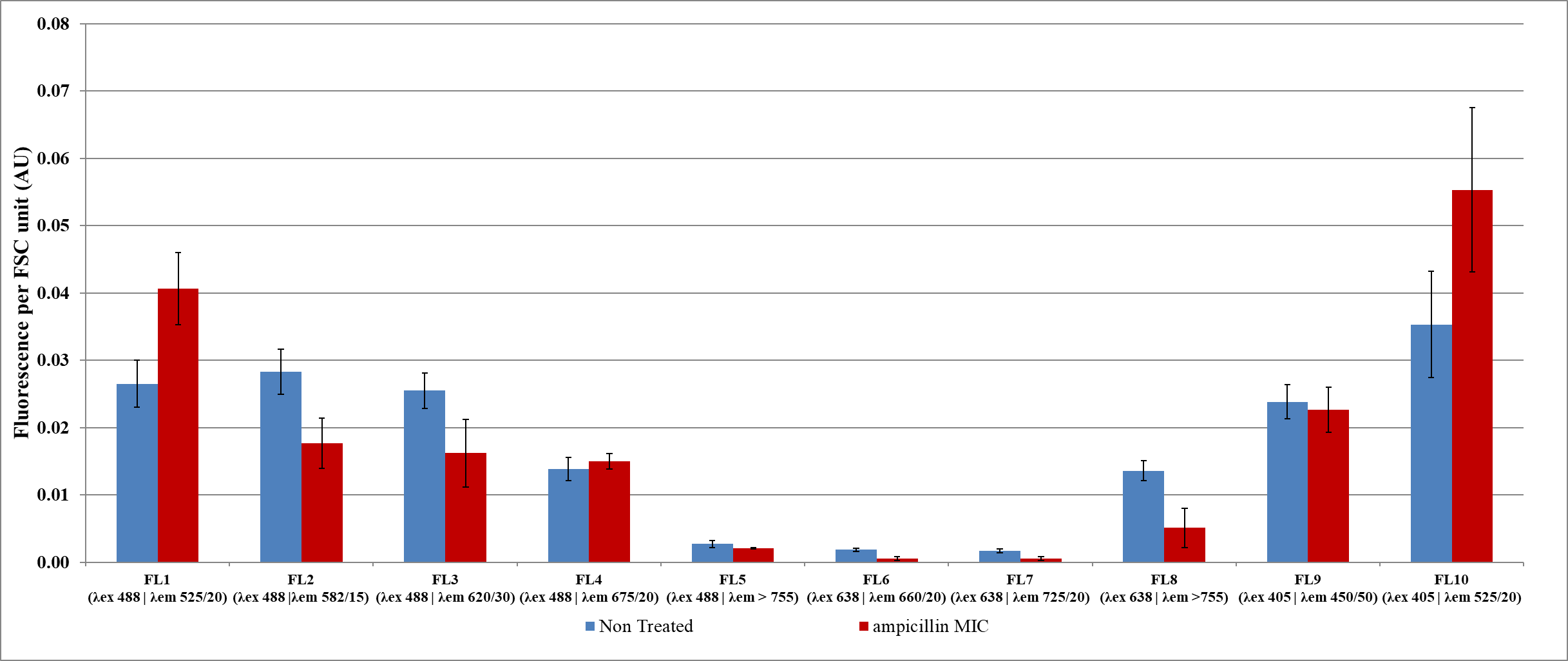 |
| --- |

**Supplemental Figure S3: *E. coli* cells’ autofluorescence signal with various excitation and emission wavelengths.** Exponentially growing *E. coli* 7705035 cells were treated with ampicillin MIC concentration (4mg/L) for 2 hours. Autofluorescence and FSC of treated and untreated controls were measured using different channels of a flow cytometer. Cell autofluorescence was normalized by the FSC signal to obtain the autofluorescence per FSC unit signal of each channel. Each value represents the mean +/- standard error of the medians of non-treated (blue), ampicillin MIC (4 mg/L) (red) conditions, from three independent experiments.

| 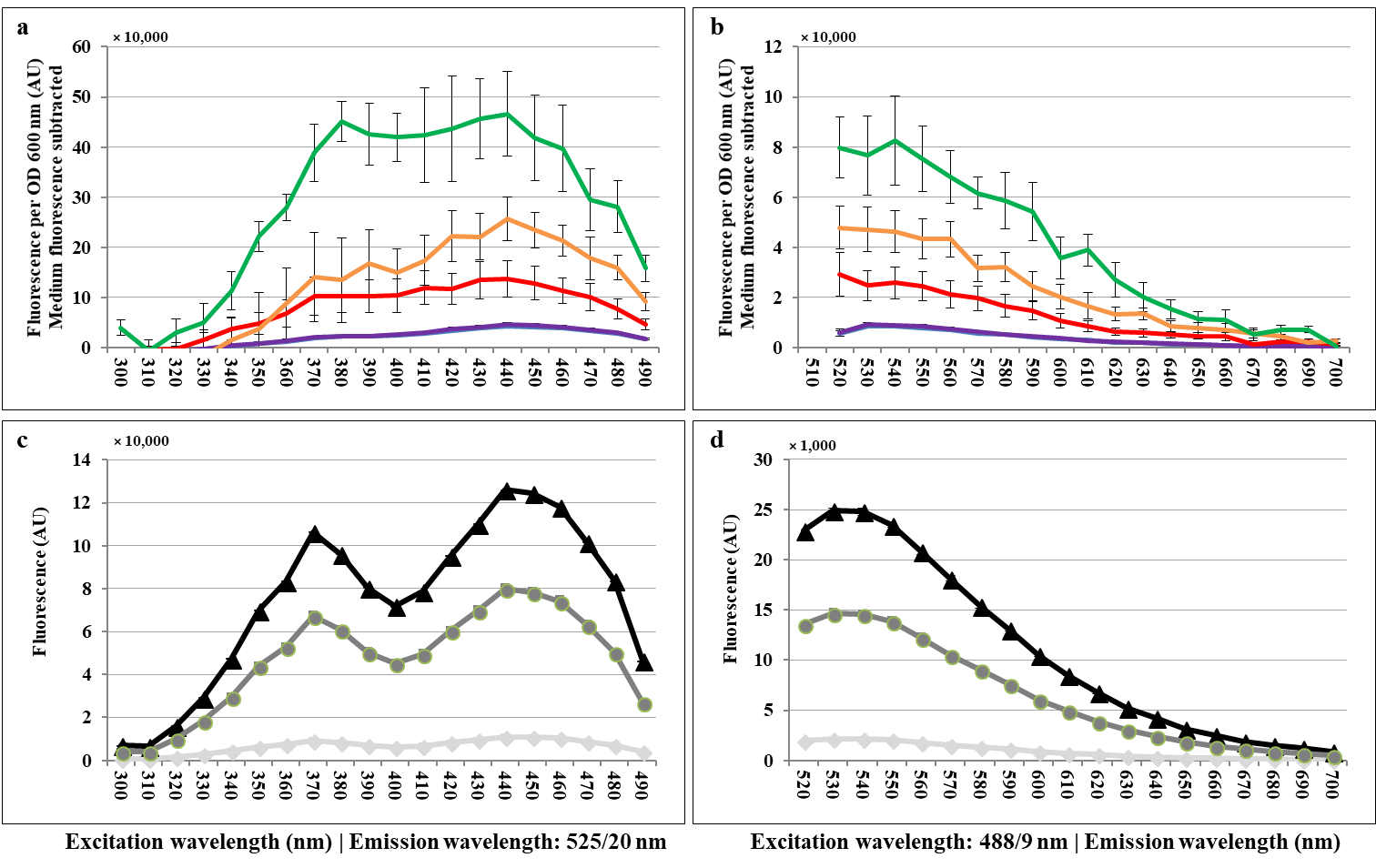 |
| --- |
| **Supplemental Figure S4: Excitation and emission spectra of *E. coli* cells and purified flavonoid compounds.** *E. coli* 7705035 cells [ampicillin MIC = 4 mg/L] growing exponentially in LB medium were treated with a range of ampicillin concentrations for 3 hours. After 3 hours of treatment, the excitation and emission spectra of treated cells were established and compared with the spectra of FAD, FMN and riboflavin. The medium background was subtracted and fluorescence spectra of *E. coli* cells were normalized to the Optical Density at 600 nm. (**a**) and (**b**) Excitation and emission spectra of *E. coli* with ampicillin concentrations: 0 (blue line), 1 (violet line), 4 (red line), 8 (yellow line) and 32 mg/L (green line). (**c**) and (**d**) Excitation and emission spectra of 10 µM purified flavonoid compounds FAD (light grey diamond), FMN (black triangle) and riboflavin (dark grey circles). Each value represents the mean +/- standard error of the fluorescence from 5 independent experiments. |

| 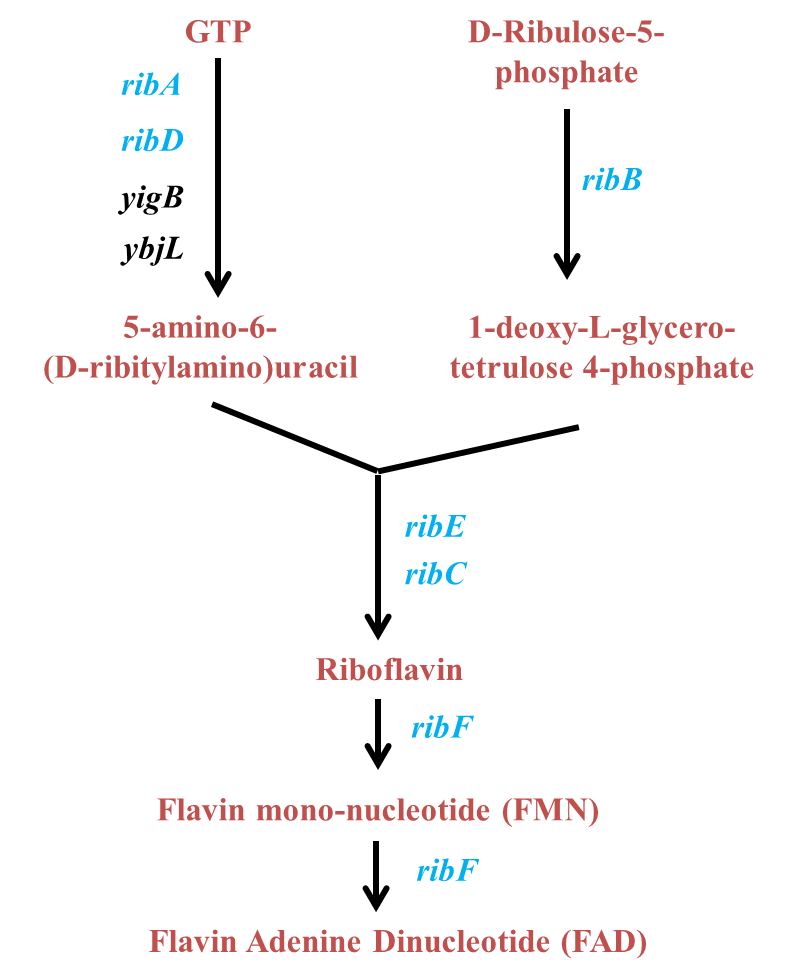 |
| --- |

**Supplemental Figure S5: Genes involved in the flavin biosynthesis pathway** (Adapted from https://ecocyc.org/). Red letters represent the metabolites in the pathway. Light blue represents essential genes in the pathway. Black represents the non-essential genes in the pathway.

**Supplemental Table S1: Strains used in the study**

| **Bacterial clinical isolates** | | | | | | | |
| --- | --- | --- | --- | --- | --- | --- | --- |
| **Species** | **Strain** | **Susceptibility to antibiotics** | | | | | **Origins / References** |
| *Escherichia coli* | 7705035 | ampicillin  MIC = 4  mg/L | | gentamicin  MIC = 0.5  mg/L | | tetracycline  MIC = 1 mg/L | ATCC strain collection.  https://www.lgcstandards-atcc.org/Products/Cells_and_Microorganisms |
| *Escherichia coli* | 8812112 | ampicillin  MIC = 64 mg/L | | | | | bioMérieux strain collection |
| *Klebsiella pneumoniae ssp pneumoniae* | 0911053 | meropenem  MIC = 16 mg/L | | | | | ibid |
| *Serratia marcescens* | 9603043 | meropenem  MIC = 2 mg/L | | | | | ibid |
|  | | | | | | | |
| ***E. coli* strains carrying plasmid-borne transcriptional reporter fusions** | | | | | | | |
| **Species** | **Relevant phenotype** | | **Promoters of the following genes were fused to *gfp*** | | **Susceptibility to antibiotics** | | **Origins / References** |
| *Escherichia coli* | KanR | | *lacZ* | | 2 mg/L | | Zaslaver A, *et al.* Nature Methods3,623-628 (2006) |
| *Escherichia coli* | KanR | | *aroC* | | 2 mg/L | | ibid |
| *Escherichia coli* | KanR | | *cspA* | | 2 mg/L | | ibid |
| *Escherichia coli* | KanR | | *cysJ* | | 2 mg/L | | ibid |
| *Escherichia coli* | KanR | | *dfp* | | 2 mg/L | | ibid |
| *Escherichia coli* | KanR | | *fldA* | | 2 mg/L | | ibid |
| *Escherichia coli* | KanR | | *fpr* | | 2 mg/L | | ibid |
| *Escherichia coli* | KanR | | *frdA* | | 2 mg/L | | ibid |
| *Escherichia coli* | KanR | | *gcl* | | 2 mg/L | | ibid |
| *Escherichia coli* | KanR | | *glpA* | | 2 mg/L | | ibid |
| *Escherichia coli* | KanR | | *glpD* | | 2 mg/L | | ibid |
| *Escherichia coli* | KanR | | *ilvI* | | 2 mg/L | | ibid |
| *Escherichia coli* | KanR | | *lpd* | | 2 mg/L | | ibid |
| *Escherichia coli* | KanR | | *metF* | | 2 mg/L | | ibid |
| *Escherichia coli* | KanR | | *mqo* | | 2 mg/L | | ibid |
| *Escherichia coli* | KanR | | *ndh* | | 2 mg/L | | ibid |
| *Escherichia coli* | KanR | | *nfnB* | | 2 mg/L | | ibid |
| *Escherichia coli* | KanR | | *norV/norW* | | 2 mg/L | | ibid |
| *Escherichia coli* | KanR | | *nuoA/nuoF* | | 2 mg/L | | ibid |
| *Escherichia coli* | KanR | | *pdxH* | | 2 mg/L | | ibid |
| *Escherichia coli* | KanR | | *poxB* | | 2 mg/L | | ibid |
| *Escherichia coli* | KanR | | *ribA* | | 2 mg/L | | ibid |
| *Escherichia coli* | KanR | | *ribB* | | 2 mg/L | | ibid |
| *Escherichia coli* | KanR | | *ribC* | | 2 mg/L | | ibid |
| *Escherichia coli* | KanR | | *ribE* | | 2 mg/L | | ibid |
| *Escherichia coli* | KanR | | *sdhA/sdhC* | | 2 mg/L | | ibid |
| *Escherichia coli* | KanR | | *soxR* | | 2 mg/L | | ibid |
| *Escherichia coli* | KanR | | *ssuD/ssuE* | | 2 mg/L | | ibid |
| *Escherichia coli* | KanR | | *uxaC* | | 2 mg/L | | ibid |
| *Escherichia coli* | KanR | | *wrbA - 2* | | 2 mg/L | | ibid |
| *Escherichia coli* | KanR | | *wrbA -1* | | 2 mg/L | | ibid |
| *Escherichia coli* | KanR | | *wrbA-3* | | 2 mg/L | | ibid |
| *Escherichia coli* | KanR | | *ycaK* | | 2 mg/L | | ibid |
| *Escherichia coli* | KanR | | *ycdH/ycdM* | | 2 mg/L | | ibid |
| *Escherichia coli* | KanR | | *yeeO* | | 2 mg/L | | ibid |
| *Escherichia coli* | KanR | | *yieF/yieE* | | 2 mg/L | | ibid |
| *Escherichia coli* | KanR | | promoter less | | 2 mg/L | | ibid |
|  | | | | | | | |
| ***E. coli* strains from KEIO collection** | | | | | | | |
| **Species** | **Strain Name** | **Relevant Genotype** | | **Susceptibility to antibiotics** | | | **Origins / References** |
| *Escherichia coli* | BW25113 | Wild-type | | ampicillin  MIC =4 mg/L | | | Baba *et. al*, Molecular Systems Biology, 2006 |
| *Escherichia coli* | *JW1965* | *∆yeeO* | | ampicillin  MIC = 4 mg/L | | | ibid |
|  | | | | | | | |
| **Eukaryotic cells** | | | | | | | |
| **Species** | **Strain/Cell line** | **Relevant Genotype** | | | | | **Origins / References** |
| *Saccharomyces cerevisiae* | *SK1* | MATa/α HO gal2 cupS can1R BIO | | | | | Angela Taddei laboratory collection, Institut Curie, Paris, France |
| Human | HeLa | Cervical cancer cells | | | | | Benoît Miotto laboratory, Institut Cochin, INSERM U1016, Paris, France ^3^ |

**Supplemental Table S2: correlation between FSC, SSC and FL1 of the ampicillin-treated cells.**

**Part A: correlation between FSC and FL1**

| **Time 1 hour** | | | **Time 2 hours** | | **Time 3 hours** | |
| --- | --- | --- | --- | --- | --- | --- |
| **amp (mg/L)** | **r value** | **p value** | **r value** | **p value** | **r value** | **p value** |
| 0 | **0.5840** | < 0.0001 | **0.3259** | < 0.0001 | **0.3277** | < 0.0001 |
| 1 | **0.6568** | < 0.0001 | **0.7175** | < 0.0001 | **0.8149** | < 0.0001 |
| 2 | **0.8036** | < 0.0001 | **0.8517** | < 0.0001 | **0.8926** | < 0.0001 |
| 4 | **0.8156** | < 0.0001 | **0.8909** | < 0.0001 | **0.9257** | < 0.0001 |
| 8 | **0.8422** | < 0.0001 | **0.8523** | < 0.0001 | **0.7853** | < 0.0001 |
| 16 | **0.8858** | < 0.0001 | **0.7197** | < 0.0001 | **0.5728** | < 0.0001 |
| 32 | **0.8679** | < 0.0001 | **0.6807** | < 0.0001 | **0.5841** | < 0.0001 |
| 64 | **0.8562** | < 0.0001 | **0.5931** | < 0.0001 | **0.5818** | < 0.0001 |

**Part B: correlation between SSC and FL1**

| **Time 1 hour** | | | **Time 2 hours** | | **Time 3 hours** | |
| --- | --- | --- | --- | --- | --- | --- |
| **amp (mg/L)** | **r value** | **p value** | **r value** | **p value** | **r value** | **p value** |
| 0 | **0.4760** | < 0.0001 | **0.2784** | < 0.0001 | **0.2453** | < 0.0001 |
| 1 | **0.5755** | < 0.0001 | **0.8142** | < 0.0001 | **0.8877** | < 0.0001 |
| 2 | **0.7596** | < 0.0001 | **0.8641** | < 0.0001 | **0.8934** | < 0.0001 |
| 4 | **0.7822** | < 0.0001 | **0.8759** | < 0.0001 | **0.9153** | < 0.0001 |
| 8 | **0.8324** | < 0.0001 | **0.6957** | < 0.0001 | **0.4534** | < 0.0001 |
| 16 | **0.8710** | < 0.0001 | **0.6777** | < 0.0001 | **0.4598** | < 0.0001 |
| 32 | **0.8233** | < 0.0001 | **0.6709** | < 0.0001 | **0.6026** | < 0.0001 |
| 64 | **0.8208** | < 0.0001 | **0.5953** | < 0.0001 | **0.6081** | < 0.0001 |

Exponentially growing *E. coli* 7705035 cells were treated with a range of ampicillin concentrations for 3 hours. For each condition, autofluorescence and light scattering signals (FSC and SSC) of 50,000 cells were measured after 1, 2 and 3 hours of incubation by flow cytometry. Correlation between FSC/FL1 and SSC/FL1 was assessed for each of the 50,000 cells.

**References**

1. Zaslaver A*, et al.* A comprehensive library of fluorescent transcriptional reporters for *Escherichia coli*. *Nat Methods* **3**, 623-628 (2006).

2. Baba T*, et al.* Construction of Escherichia coli K-12 in-frame, single-gene knockout mutants: the Keio collection. *Molecular systems biology* **2**, 2006 0008 (2006).

3. Scherer WF, Syverton JT, Gey GO. Studies on the propagation in vitro of poliomyelitis viruses. IV. Viral multiplication in a stable strain of human malignant epithelial cells (strain HeLa) derived from an epidermoid carcinoma of the cervix. *J Exp Med* **97**, 695-710 (1953).
